# Design, synthesis, and anti-inflammatory activity of 2H-1,4-benzoxazin-3(4H)-one derivatives modified with 1,2,3-triazole in LPS-induced BV-2 cells

**DOI:** 10.3389/fphar.2024.1509520

**Published:** 2025-01-20

**Authors:** Xixi Hou, Longfei Mao, Huibin Zhang, Lan Wang, Baoyu He, Jingjing Guo, Jianji Wang

**Affiliations:** ^1^ Key Laboratory of Green Chemical Media and Reactions, Ministry of Education, Collaborative Innovation Center of Henan Province for Green Manufacturing of Fine Chemicals, School of Chemistry and Chemical Engineering, Henan Normal University, Xinxiang, Henan, China; ^2^ The First Affiliated Hospital, College of Clinical Medicine of Henan University of Science and Technology, Luoyang, Henan, China; ^3^ College of Basic Medicine and Forensic Medicine, Henan University of Science and Technology, Luoyang, Henan, China; ^4^ Centre for Artificial Intelligence Driven Drug Discovery, Faculty of Applied Sciences, Macao Polytechnic University, Macao, China

**Keywords:** 1,2,3-triazoles, 2H-1,4-benzoxazin-3(4H)-one, microglial cells, LPS-induced, antiinflammatory

## Abstract

Given the potent anti-inflammatory properties of the 1,2,3-triazole structure and the wide use of 2H-1,4-benzoxazin-3(4H)-one in developing treatments for neurodegenerative diseases, a series of 2H-1,4-benzoxazin-3(4H)-one derivatives were synthesized by introducing a 1,2,3-triazole moiety. Screening for anti-inflammatory activity in microglial cells revealed that compounds e2, e16, and e20 exhibited the most promising effects without significant cytotoxicity. These compounds effectively reduced LPS-induced NO production and significantly decreased the transcription levels of pro-inflammatory cytokines IL-1β, IL-6, and TNF-α. Furthermore, they downregulated the transcription and protein levels of the inflammation-related enzymes iNOS and COX-2 in response to LPS stimulation. To further investigate the anti-inflammatory mechanisms of these derivatives in microglia, the intracellular ROS levels and the activation of the Nrf2-HO-1 signaling pathway were analyzed. The results indicated that the 2H-1,4-benzoxazin-3(4H)-one derivatives significantly activated the Nrf2-HO-1 pathway, reduced LPS-induced ROS production, and alleviated microglial inflammation. Molecular docking studies suggested that compounds e2, e16, and e20 could interact with Nrf2-related binding sites, preventing its degradation by Keap1. Additionally, acute toxicity tests in mice demonstrated that compound e16 exhibited favorable safety.

## 1 Introduction

Microglial inflammation is a hallmark of brain injury and plays a significant role in the pathogenesis of neurodegenerative diseases such as depression, Alzheimer’s disease, Parkinson’s disease, and multiple sclerosis (Yoon et al., 2023). As the key immune cells in the central nervous system (CNS), the primary physiological function of microglia is to regulate the brain’s immune defense mechanisms (Sasimol et al., 2018). When activated by lipopolysaccharide (LPS), microglia increase intracellular reactive oxygen species (ROS), leading to oxidative stress responses. This induces the production of intracellular inducible nitric oxide synthase (iNOS) and cyclooxygenase-2 (COX-2), promoting the release of large amounts of inflammatory factors such as nitric oxide (NO), interleukin-1β (IL-1β), interleukin-6 (IL-6), and tumor necrosis factor-α (TNF-α), subsequently causing neuronal damage (Zhu et al., 2021). Numerous studies have shown that depression is accompanied by the activation of the inflammatory response system, with changes in pro-inflammatory cytokine levels in the brains of depressed patients, such as increased secretion of interleukins (IL) and tumor necrosis factor (TNF), particularly elevated levels of IL-1β, IL-6, and TNF-α (Jung et al., 2023; Li et al., 2024). Therefore, inhibiting microglial inflammation may be a potential method for treating neuroinflammatory diseases like depression.

1,2,3-Triazole is a very important class of nitrogen-containing five-membered aromatic heterocyclic compounds, which can serve as functional heterocyclic compounds, be embedded in polycyclic structures, or be used to connect multiple active molecules (Guan et al., 2024). Its planar rigid structure has a π-π conjugated system and a large dipole moment (μ), making it prone to non-covalent interactions (Cidade et al., 2021), such as hydrophobic effects, electrostatic interactions, van der Waals forces, and hydrogen bonding. These interactions endow it with good biological functions, enabling it to bind with various enzymes and receptors in the body (Mao et al., 2020), exhibiting a wide range of pharmacological activities including anti-cancer, antiviral, anti-inflammatory, antibacterial, and antimalarial properties (Hou et al., 2024; Xu et al., 2019; Begam et al., 2022; Liu et al., 2024). 1,2,3-Triazole can act as an electronic isostere for structures like amides (Chen et al., 2022; He et al., 2020), esters, carboxylic acids, and alkenes, making it an important drug molecule building block for the development of innovative drugs (Kim et al., 2015; Xie et al., 2023).

**FIGURE 1:**
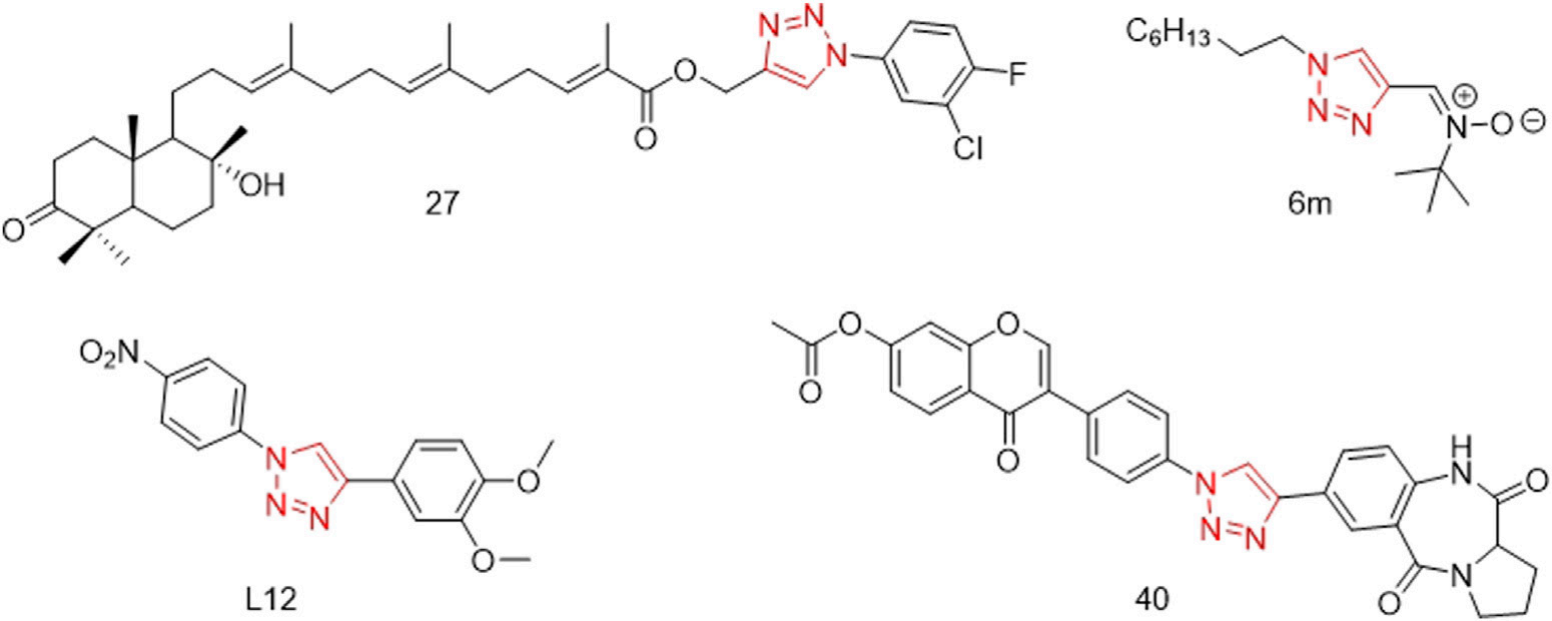
Structures of compound 27, compound 6m, compound L12 and compound 40 (the red is 1,2,3-triazole structure).

Some compounds with a 1,2,3-triazole structure can inhibit the activity or secretion function of inflammatory factors, thereby exerting anti-inflammatory effects and potentially serving as drugs for treating inflammation-related diseases (Shafi et al., 2012). For example, Madasu’s research group used guggulsterone B as the core structure and introduced a 1,2,3-triazole group via a Cu-catalyzed Huisgen 1,3-dipolar cycloaddition reaction. These compounds not only exhibit excellent antitumor activity but also possess good anti-inflammatory activity. Among them, compound **27** (Figure 1) significantly inhibited the inflammatory cytokines TNF-α (IC_50_: 7.83 ± 0.95 µM) and IL-1β (IC_50_: 15.84 ± 0.82 µM), outperforming guggulsterone B (Madasu et al., 2020). Rao’s research group designed and synthesized a series of novel 1,2,3-triazole-substituted N-phenyl isoxazolone derivatives (6a-6p) and screened them for anti-inflammatory activity. Among these, compound **6m** (Figure 1) showed significant inhibition of IL-1β secretion (IC_50_: 7.9 ± 1.36 µM) (Sambasiva Rao et al., 2014). Felipe’s research group designed and synthesized a series of compounds containing 1,2,3-triazole based on the structures of neolignans and celecoxib. Among these, compound **L12** (Figure 1) exhibited excellent anti-inflammatory activity. Molecular docking studies revealed that the binding mode of compound **L12** at the COX-2 active site was similar to that of celecoxib (docking score: −70.387 KJ/mol), indicating its potential as a COX-2 inhibitor (Felipe et al., 2022). Mengheres’ group used a copper-catalyzed azide-alkyne cycloaddition reaction to obtain eight novel 1,2,3-triazole-linked isoflavone-benzodiazepine compounds. The evaluation of their anti-inflammatory activity by inhibiting cell viability and NO production in LPS-induced BV-2 cells showed that some compounds exhibited superior NO inhibitory activity compared to natural isoflavones, with compound **40** (Figure 1) reducing NO release by 64.28% without affecting BV-2 cell viability (Menghere et al., 2020).

**FIGURE 2:**
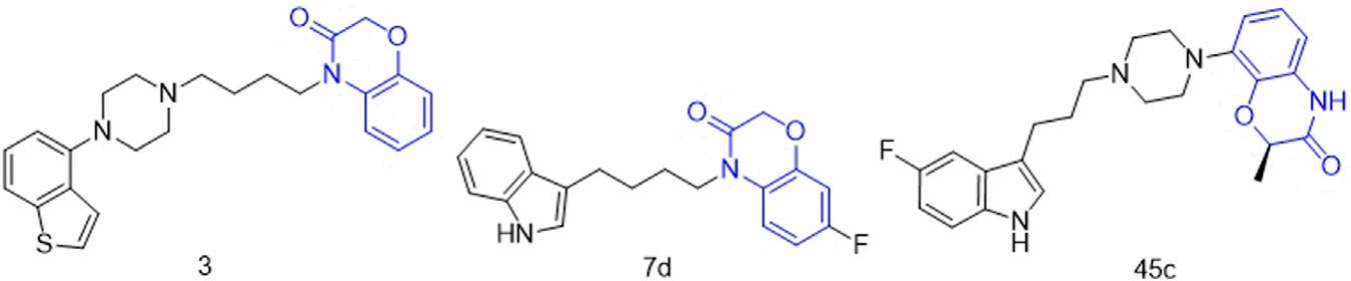
Structures of compound 3, compound 7d and compound 45c (the green is 2H-1,4-Benzoxazin-3(4H)-one structure).

2H-1,4-Benzoxazin-3(4H)-one is an important nitrogen and oxygen-containing heterocyclic compound with broad biological activity and relatively low biological toxicity (Hasui et al., 2011; Zhou et al., 2014; Yan et al., 2019; Sagam et al., 2024). A large number of derivatives based on this core structure have been synthesized, especially for use in research related to psychiatric diseases. Jiang’s research group constructed an artificial intelligence drug generation system capable of targeting multiple G protein-coupled receptors (GPCRs) using deep recurrent neural networks (RNN) and multi-task deep neural networks (MTDNN). They discovered that compound **3** (Figure 2), which has a 2H-1,4-Benzoxazin-3(4H)-one scaffold, exhibited strong activity against dopamine D2 receptors, serotonin 5-HT1A, and 5-HT2A receptors, making it a potential lead compound for the treatment of complex neuropsychiatric disorders [28]. Claudio’s research group designed and synthesized compound **7d** (Figure 2), which has a 2H-1,4-Benzoxazin-3(4H)-one scaffold and can non-competitively inhibit human acetylcholinesterase (hAChE) activity with a Ki value of 20.2 ± 0.9 μM, indicating its potential as a treatment for Alzheimer’s disease (Méndez-Rojas et al., 2018). Smid’s research group designed compound **45c** (Figure 2), which also has a 2H-1,4-Benzoxazin-3(4H)-one scaffold. It can act as a potent dopamine D2 receptor antagonist and exhibits high activity in inhibiting serotonin reuptake, suggesting its potential for the treatment of depression.

Given the excellent anti-inflammatory properties of the 1,2,3-triazole structure and the widespread application of 2H-1,4-Benzoxazin-3(4H)-one in the development of drugs for neurodegenerative diseases, we propose to structurally modify 2H-1,4-Benzoxazin-3(4H)-one with a 1,2,3-triazole moiety. This modification aims to investigate whether it can impart or enhance anti-inflammatory activity in BV2 neural cells. By doing so, we hope to discover lead compounds with outstanding anti-inflammatory properties that can be further developed into drugs for treating neurodegenerative diseases.

## 2 Chemistry

**FIGURE 3:**
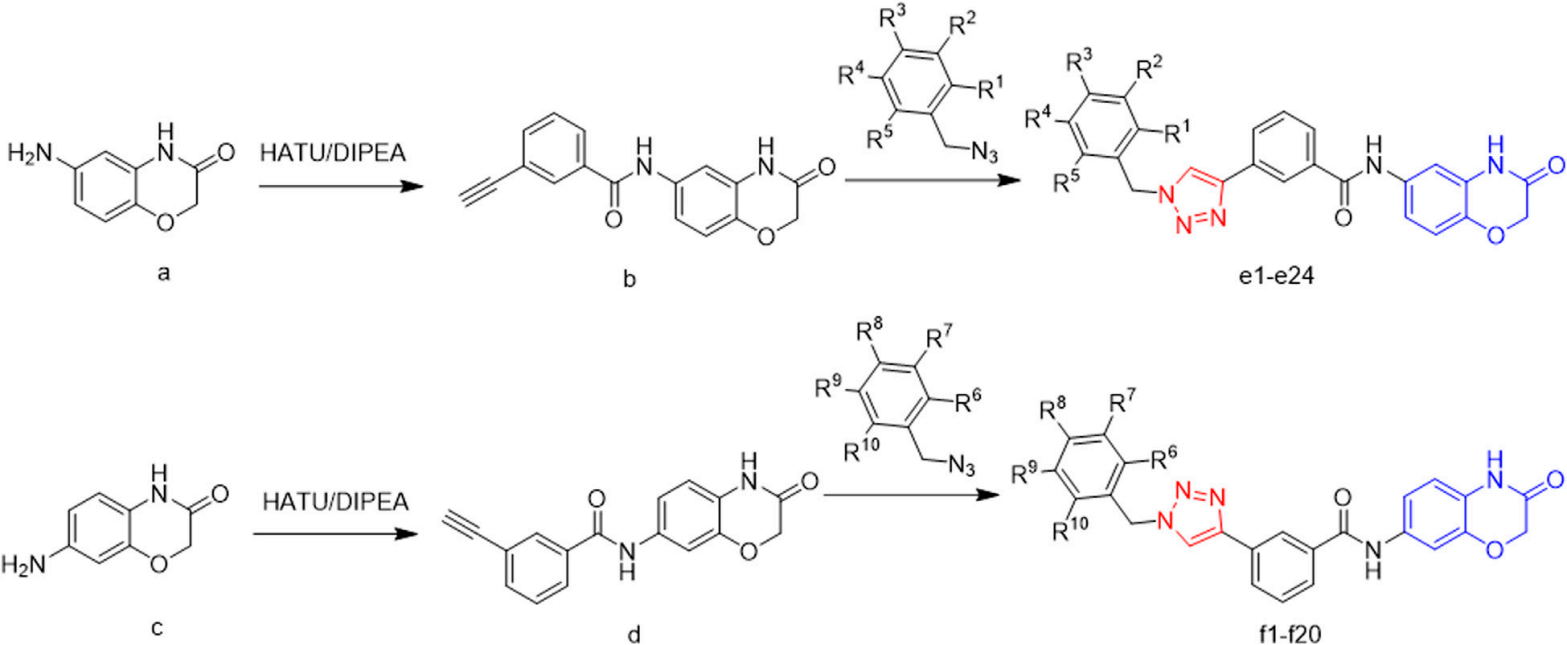
The reaction routes to compounds e1-e24 and j1-j20.

In this route, 6-amino-2H-benzo[b][1,4]oxazin-3(4H)-one (**a**) was used as raw material and it reacted with 3-ethynylbenzoic acid under the action of HATU and DIPEA to obtain 3-ethynyl-N-(3-oxo-3,4-dihydro-2H-benzo[b][1,4]oxazin-6-yl)benzamide (**b**). Compound **b** was reacted with azide compounds of different substituents to obtain 24 novel structure target compounds (**e1-e24**) as shown in Figure 3 and Table 1. Compounds (**f1-f24**) were prepared using the same method as shown in Figure 3 and Table 2. The structures of the target compound were confirmed through ^1^H and ^13^C nuclear magnetic resonance (^1^H NMR and ^13^C NMR) spectroscopy.

**TABLE 1:** R-group of compounds e1-e24.

Compounds	R^1^	R^2^	R^3^	R^4^	R^5^
e1	H	H	Br	H	H
e2	H	CH_3_	H	CH_3_	H
e3	H	F	F	F	H
e4	NC	H	H	F	H
e5	F	H	H	H	Cl
e6	H	Cl	F	H	H
e7	H	Cl	H	H	H
e8	F	H	H	Cl	H
e9	H	H	F	H	H
e10	F	H	H	H	H
e11	H	Br	H	Br	H
e12	F	H	Br	H	H
e13	H	CF_3_	H	H	H
e14	Cl	H	H	H	Cl
e15	NC	H	H	H	H
e16	H	H	CF_3_	H	H
e17	Br	H	H	H	H
e18	H	F	H	H	H
e19	H	H	I	H	H
e20	NO_2_	H	H	H	H
e21	H	H	Cl	H	H
e22	CF_3_	H	H	H	H
e23	H	H	H	H	H
e24	Cl	H	H	F	H

**TABLE 2:** R-group of compounds f1–f20.

Compounds	R^6^	R^7^	R^8^	R^9^	R^10^
f1	Cl	H	H	H	Cl
f2	H	Cl	H	H	H
f3	F	H	H	H	H
f4	H	H	I	H	H
f5	H	H	Br	H	H
f6	H	CH_3_	H	CH_3_	H
f7	CN	H	H	H	H
f8	H	CF_3_	H	H	H
f9	H	Cl	F	H	H
f10	F	H	H	H	F
f11	CN	H	H	F	H
f12	Br	H	H	H	H
f13	F	H	Br	H	H
f14	H	F	H	H	H
f15	Cl	H	H	H	F
f16	H	H	CF_3_	H	H
f17	H	Br	H	H	H
f18	H	H	Cl	H	H
f19	H	I	H	H	H
f20	CH_3_	H	H	H	H

## 3 Results

### 3.1 2H-1,4-benzoxazin-3(4H)-one derivatives inhibited LPS-induced NO production in BV-2 cells

To assess the anti-inflammatory activities of 2H-1,4-Benzoxazin-3(4H)-one derivatives, the NO levels in the supernatant of BV-2 microglial cells were measured using the Griess method. Resveratrol was used as a positive control, and 20 μM of resveratrol reduced the NO levels in the cell supernatant to 42.02% ± 2.50% of the LPS group. As shown in Table 3, the LPS-induced NO production in BV-2 cells aftertreated by 2H-1,4-Benzoxazin-3(4H)-one derivatives at a concentration of 10 μM are summarized. Additionally, the effects of these compounds on the viability of BV-2 microglial cells were assessed using the MTT assay. Based on the above data, a structure-activity relationship analysis of the compounds was conducted. By comparing the activity data of compounds with the same substituents in the e and j series, such as **e1** and **f5**, **e2** and **f6**, **e6** and **f9**, **e7** and **f2**, **e12** and **f13**, **e13** and **f8**, **e16** and **f16**, **e19** and **f4**, and the terminal alkyne intermediates **b** and **d**, we can clearly observe that the e series compounds are more effective at reducing NO levels in BV-2 cells and exhibit no significant cytotoxicity in BV-2 cells, especially compounds **e2**, **e16**, and **e20**, which show no obvious cytotoxicity and have the best anti-inflammatory effects.

**TABLE 3:** The inhibitory effects of the 2H-1,4-Benzoxazin-3(4H)-one derivatives on LPS-induced NO production in BV-2 cells.

Compounds	NO production^a^	Cell viability^b^	Compounds	NO production^a^	Cell viability^b^
e1	60.91 ± 11.84*	90.68 ± 3.68	f1	70.51 ± 4.29**	50.48 ± 2.52^###^
e2	54.66 ± 8.93**	101.60 ± 1.48	f2	103.1 ± 2.59	47.01 ± 3.02^###^
e3	62.37 ± 4.89**	72.95 ± 11.47	f3	79.55 ± 11.36	51.24 ± 1.96^###^
e4	88.31 ± 8.26	86.55 ± 3.09^#^	f4	100.4 ± 8.81	47.66 ± 2.84^###^
e5	92.08 ± 10.49	44.11 ± 8.32^##^	f5	101.1 ± 1.64	45.87 ± 5.42^###^
e6	57.05 ± 9.11**	74.52 ± 3.10^##^	f6	94.63 ± 9.73	56.96 ± 3.34^###^
e7	64.67 ± 6.27**	74.4 ± 8.64^#^	f7	95.49 ± 2.00	89.51 ± 1.50^##^
e8	90.73 ± 13.31	83.56 ± 15.64	f8	105.8 ± 6.01	61.27 ± 4.61^###^
e9	80.16 ± 4.71*	75.57 ± 2.27^###^	f9	103.6 ± 5.07	62.99 ± 2.67^###^
e10	81.90 ± 6.33*	73.86 ± 2.26^###^	f10	79.67 ± 7.00*	68.51 ± 1.10^###^
e11	44.44 ± 3.66***	44.05 ± 2.31^###^	f11	100.9 ± 8.62	68.16 ± 3.81^###^
e12	71.74 ± 4.39**	101.1 ± 1.95	f12	58.55 ± 6.08**	55.99 ± 8.19^##^
e13	69.36 ± 9.52*	80.31 ± 11.92	f13	98.17 ± 12.10	44.94 ± 2.49^###^
e14	26.17 ± 3.53***	52.21 ± 3.79^###^	f14	79.52 ± 5.91*	47.69 ± 5.03^###^
e15	75.72 ± 1.30***	83.77 ± 6.27	f15	89.12 ± 3.81*	68.88 ± 10.66^#^
e16	59.23 ± 4.36***	88.23 ± 10.66	f16	87.54 ± 5.32	52.45 ± 6.93^##^
e17	36.45 ± 6.21***	45.97 ± 7.44^##^	f17	88.45 ± 7.00	49.12 ± 5.16^###^
e18	72.85 ± 2.67***	62.49 ± 2.08^###^	f18	101.50 ± 3.56	45.38 ± 4.67^###^
e19	66.38 ± 14.44	103.2 ± 1.72	f19	91.53 ± 6.46	54.73 ± 0.856^###^
e20	59.19 ± 3.28***	104.1 ± 2.70	f20	105.9 ± 5.87	55.97 ± 3.84^###^
e21	87.61 ± 2.49	97.90 ± 6.42	b	65.61 ± 3.59***	75.99 ± 6.59#
e22	67.2 ± 7.12	45.48 ± 2.91	d	92.26 ± 8.95	87.95 ± 8.82
e23	91.99 ± 5.43	89.02 ± 8.41			
e24	77.65 ± 3.47	74.02 ± 5.01			
Positive control- Resveratrol (20 μM)	42.02 ± 2.50***				

*P < 0.05, **P < 0.01, ***P < 0.001 vs. the LPS, group, ^#^ *P* < 0.05, ^##^ *P* < 0.01, ^###^ *P* < 0.001 vs. the control group; n = 3.

^a^ NO data were normalized by mean value of LPS, group, which was set to 100%.

^b^ Cell viability data were normalized by mean value of control group, which was set to 100%.

### 3.2 2H-1,4-benzoxazin-3(4H)-one derivatives downregulated LPS-induced pro-inflammatory cytokine mRNA levels in BV2 microglial cells

**FIGURE 4:**
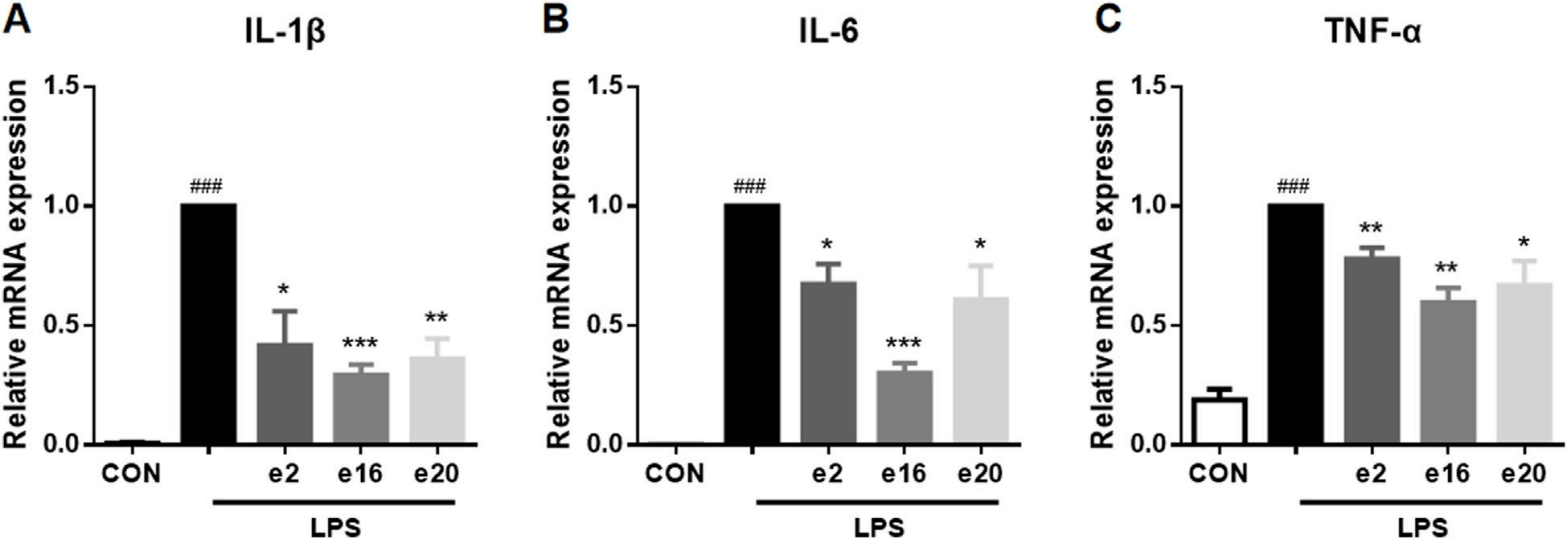
The 2H-1,4-Benzoxazin-3(4H)-one derivatives downregulated the mRNA levels of pro-inflammatory cytokines in LPS-induced BV-2 microglial cells. **(A–C)** The mRNA levels of IL-1β **(A)**, IL-6 **(B)**, and TNF-α **(C)** were measured by real-time PCR (n = 3). Data are presented as mean ± SEM. ^###^ *P* < 0.001 compared to the CON group; *P < 0.05, **P < 0.01, ***P < 0.001 compared to the LPS group.

Under LPS induction, the transcription levels of pro-inflammatory factors such as IL-1β, IL-6, and TNF-α in BV-2 microglial cells were significantly increased. To further determine the inhibitory activity of **e2**, **e16**, and **e20** on LPS-induced inflammation in microglial cells, the transcription levels of these pro-inflammatory factors in BV-2 microglial cells were measured using Real-time PCR. The results showed that **e2**, **e16**, and **e20** at a concentration of 10 μM significantly downregulated the mRNA levels of the pro-inflammatory factors IL-1β, IL-6, and TNF-α in LPS-induced microglial cells, as shown in Figure 4. This indicates that the aforementioned 2H-1,4-Benzoxazin-3(4H)-one derivatives inhibited the transcription of several key cellular inflammatory factors in LPS-induced BV-2 cells.

### 3.3 2H-1,4-benzoxazin-3(4H)-one derivatives downregulated LPS-induced iNOS and COX-2 expression levels in BV2 microglial cells

**FIGURE 5:**
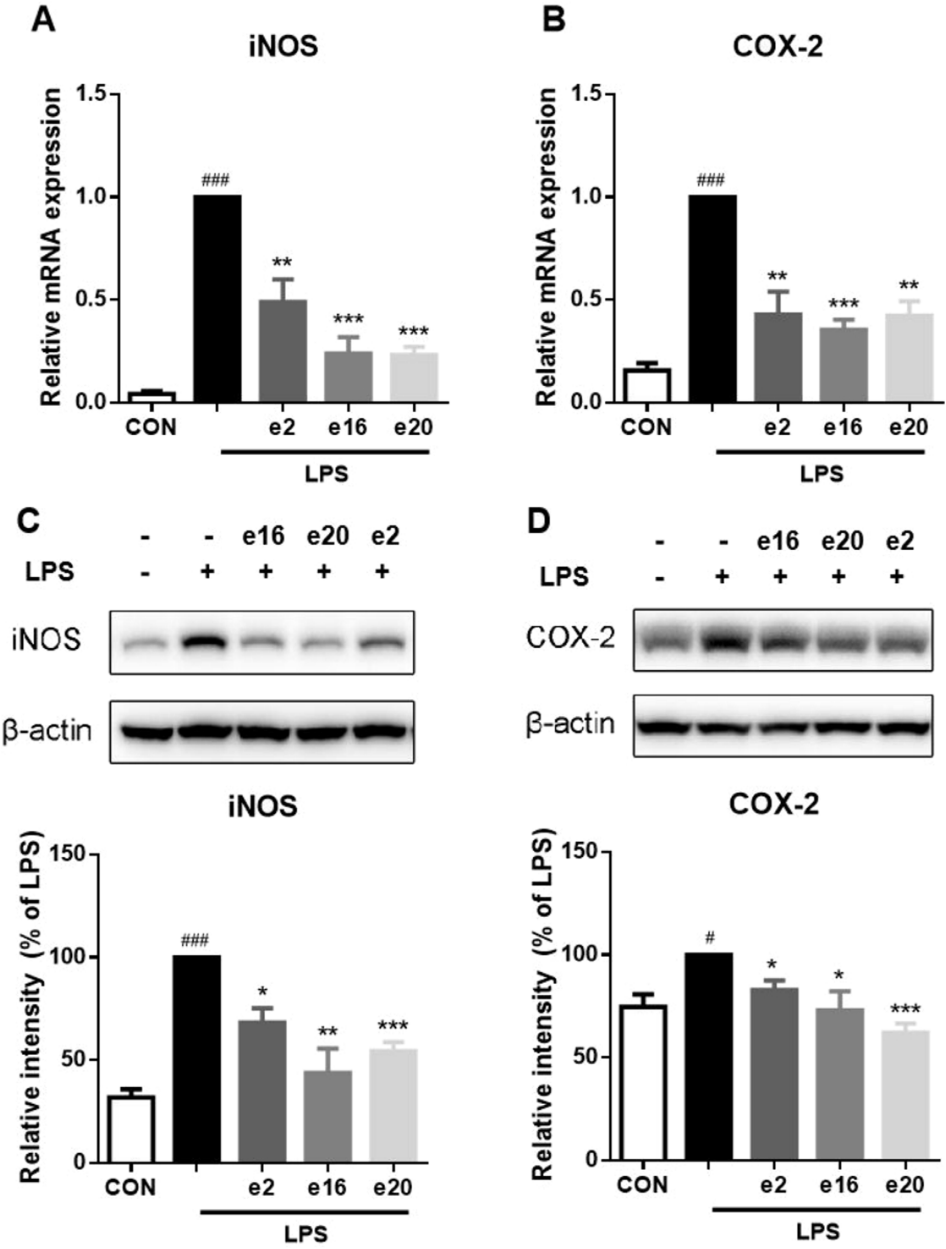
2H-1,4-Benzoxazin-3(4H)-one derivatives downregulated LPS-induced iNOS and COX-2 transcription and protein levels in BV2 microglial cells. **(A,B)** The mRNA levels of iNOS **(A)** and COX-2 **(B)** were measured by real-time PCR (n = 3). **(C,D)** The protein levels of iNOS **(C)** and COX-2 **(D)** were measured by western blot (n = 3). Data in the figure are presented as mean ± SEM. ^#^ *P* < 0.05, ^###^ *P* < 0.001 compared to the CON group; *P < 0.05, **P < 0.01, ***P < 0.001 compared to the LPS group.

The expression of inflammation-related enzymes, such as iNOS and COX-2, is also an important indicator of inflammatory effects. Therefore, the impacts of 2H-1,4-Benzoxazin-3(4H)-one derivatives on the transcription and protein levels of iNOS and COX-2 were further investigated. Under LPS treatment, the transcription levels of iNOS and COX-2 in BV-2 microglial cells significantly increased, along with corresponding increases in protein levels. However, after treatment with 10 μM e2, e16, and e20 respectively, both the transcription and protein levels of iNOS and COX-2 were significantly downregulated, as shown in Figure 5. This indicates that 2H-1,4-Benzoxazin-3(4H)-one derivatives can significantly reduce the levels of iNOS and COX-2 in BV-2 cells induced by LPS.

### 3.4 2H-1,4-benzoxazin-3(4H)-one derivatives reduced lps-induced ROS accumulation in BV-2 microglial cells

**FIGURE 6:**
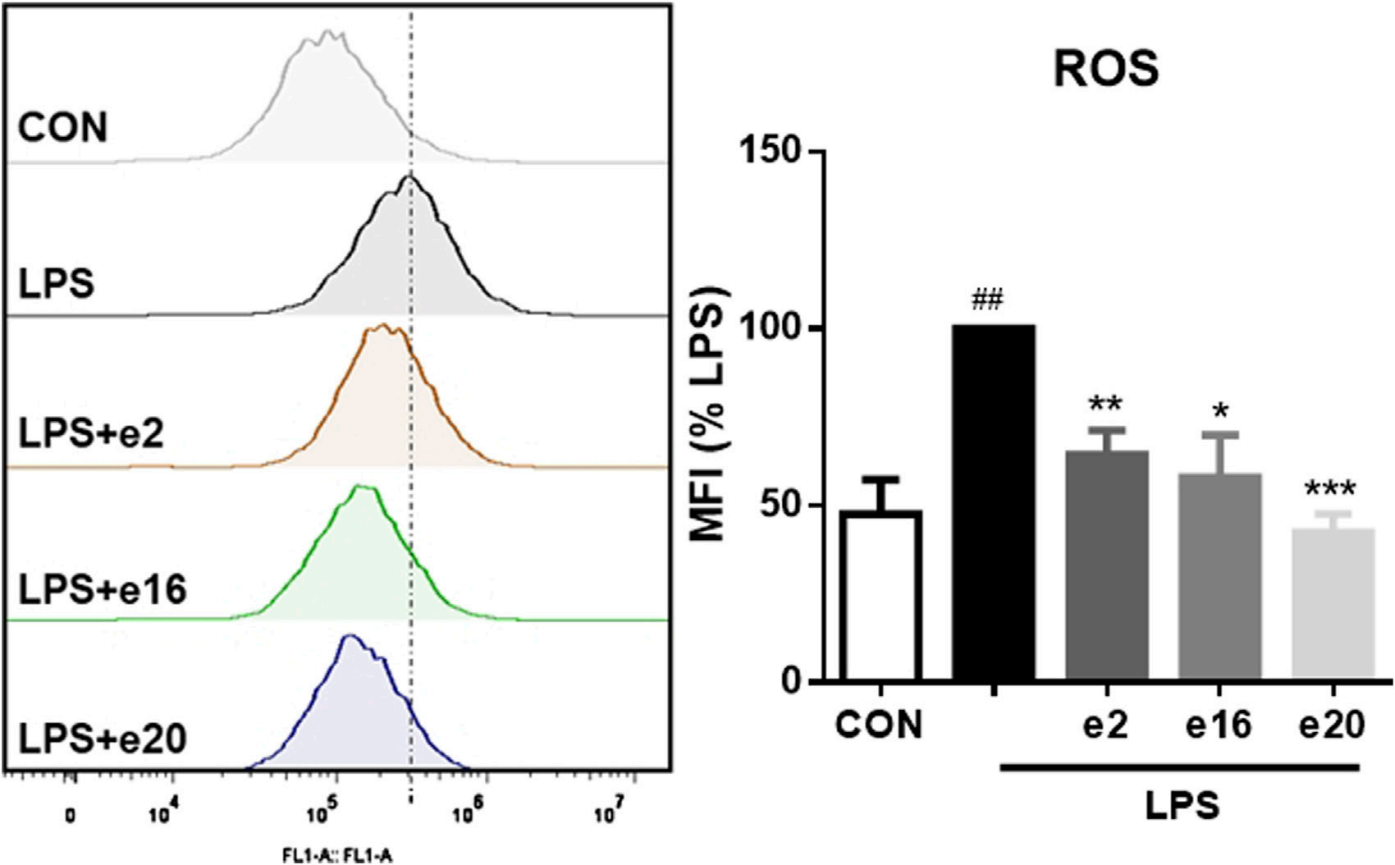
2H-1,4-Benzoxazin-3(4H)-one Derivatives downregulated LPS-induced ROS levels in BV-2 microglial cells (n = 3). Data are presented as mean ± S.E.M. ^##^ *P* < 0.01 compared to the CON group; *P < 0.05, **P < 0.01, ***P < 0.001 compared to the LPS group.

Oxidative stress can promote inflammation in microglial cells. Therefore, the regulatory effects of 2H-1,4-Benzoxazin-3(4H)-one derivatives on intracellular reactive oxygen were further investigated. The intracellular ROS levels were measured using flow cytometry, with DCFH-DA probes reflecting the ROS content within cells. After LPS treatment, ROS levels in BV-2 microglial cells significantly increased, whereas treatment with 10 μM e2, e16, and e20 significantly reduced intracellular ROS levels to 64.25% ± 6.96%, 57.90% ± 12.04%, and 42.56% ± 5.19% of the LPS group, respectively, as shown in Figure 6. This indicates that 2H-1,4-Benzoxazin-3(4H)-one derivatives can alleviate LPS-induced oxidative stress in BV-2 cells.

### 3.5 2H-1,4-benzoxazin-3(4H)-one derivatives promoted activation of the Nrf2-HO-1 pathway

**FIGURE 7:**
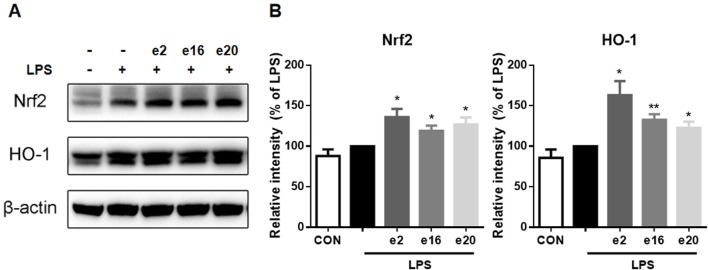
2H-1,4-Benzoxazin-3(4H)-one derivatives increased Nrf2-HO-1 protein levels in LPS-induced BV-2 Microglial Cells (n = 3). Data are presented as mean ± S.E.M. *P < 0.05, **P < 0.01 compared to the LPS group.

To further clarify the mechanism by which 2H-1,4-Benzoxazin-3(4H)-one derivatives regulated microglial cell inflammation, the Nrf2-HO-1 signaling pathway involved in oxidative stress regulation was examined. In the LPS group, Nrf2 protein levels showed no significant change. However, after treatment with 10 μM 2H-1,4-Benzoxazin-3(4H)-one derivatives, Nrf2 protein levels significantly increased. Additionally, the downstream HO-1 protein levels were also assessed (Figure 7). The results were consistent with those of Nrf2, showing that 10 μM e2, e16, and e20 significantly increased HO-1 protein levels. These results indicated that 2H-1,4-Benzoxazin-3(4H)-one derivatives could significantly activate the antioxidant Nrf2-HO-1 signaling pathway to reduce intracellular ROS levelsand thereby exert antioxidant effects to mitigate LPS-induced inflammatory effects in microglial cells.

### 3.6 Predicted binding modes of e2, e16, and e20 with Keap1

**FIGURE 8:**
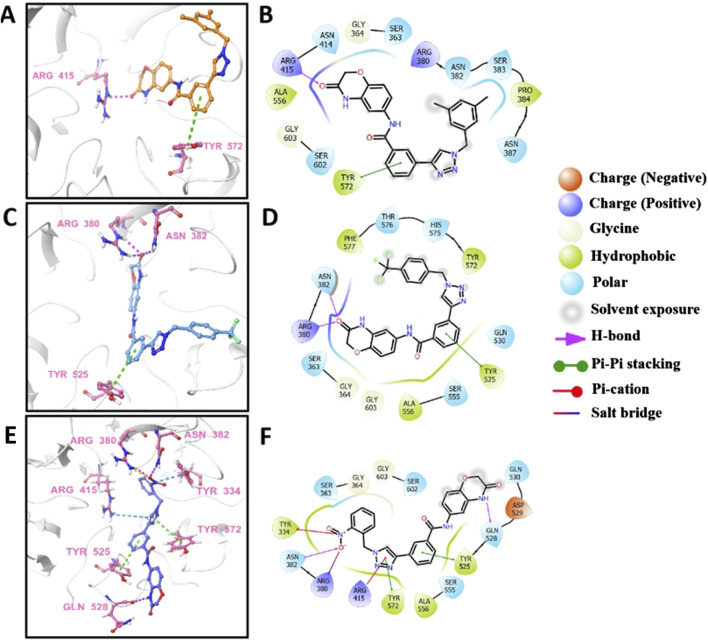
The predicted 3D models and schematic 2D diagrams illustrate the interactions between ligands and Keap1. The protein is represented as a white cartoon, while the ligands are depicted as sticks. Magenta dashed lines indicate hydrogen bonds, green dashed lines indicate pi-pi stacking, blue dashed lines indicate salt bridge, and yellow dashed lines indicate salt bridge. **(A,C, E)** the 3D diagrams of the Keap1-e2 complex, Keap1-e16 complex, and Keap1-e20 complex, respectively. **(B,D, F)** the 2D diagrams representing the interactions of ligand molecules with the amino acid residues at the binding site: **(B)** Keap1-e2 complex, **(D)** Keap1-e16 complex, and **(F)** Keap1-e20 complex.

Molecular docking is a prevalent method for predicting reliable binding modes between ligands and target proteins. Based on the aforementioned results, e2, e16, and e20 may target Keap1 to prevent Nrf2 binding. To understand the binding modes, molecular docking was employed to predict the binding interactions between these three small molecules and the Keap1 protein. As shown in Figure 8, the three molecules engage in key electrostatic interactions with important arginine residues of Keap1. In Figures 8A, B, the aldehydes of e2 form a hydrogen bond with Arg 415, while the benzene ring is involved in pi-pi stacking interactions with Tyr 572. The aldehydes of e16 form two hydrogen bonds with Arg 380 and Asn 382, and the benzene ring engages in pi-pi stacking with Tyr 525 (Figures 8C, D). The nitrobenzene of e20 forms a pi-cation interaction, a hydrogen bond, and a salt bridge with Tyr 334, Asn 382, and Arg 380, respectively. Additionally, the 1,2,3-triazoles share pi-cation and pi-pi stacking interactions with Arg 415 and Tyr 572, while the benzene ring participates in pi-pi stacking with Tyr 525 (Figures 8E, F). It has been demonstrated that the presence of an acidic moiety is essential for achieving potent activity in a lead compound (Davies et al., 2016). Therefore, e2, e16, and e20 may be potential molecules to prevent the binding of Keap1 and Nrf2 by occupying the binding site of Nrf2.

### 3.7 The acute toxicity test of compound e16 in mice

We further evaluated the safety of compound **e16** through an acute toxicity study in KM mice. Male and female mice were divided into two groups: a control group and an **e16**-treated group. The treatment group received 800 mg/kg of compound **e16** via oral gavage, while the control group was given an equivalent volume of solvent. Body weight was monitored over a 15-day period. At the end of the study, blood samples were collected from the retro-orbital sinus to assess the biochemical markers GPT and GOT, and the major organs—heart, liver, spleen, lungs, and kidneys—were weighed and subjected to HE staining.

**FIGURE 9:**
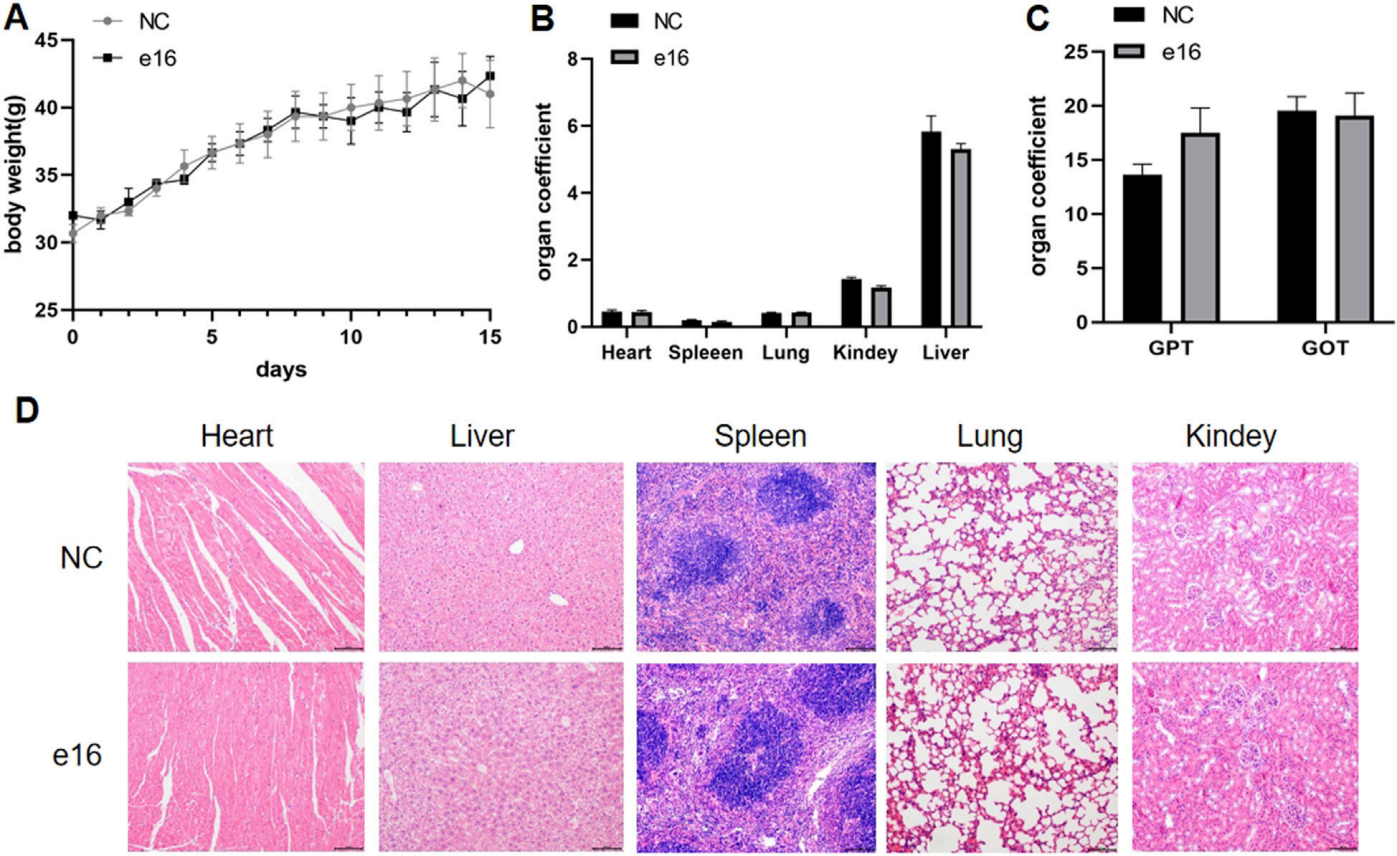
Acute toxicity experiments of compound **e16** were conducted in mice. **(A)** The body wight of mice were recorded for 15 days after treatment. **(B)** The acute toxicity experiments examined the effects of **e16** on mouse organs. **(C)** Effect of acute toxicity experimental studies on blood biochemical indices GPT and GOT in mice. **(D)** H&E staining were performed on various organs of mice treated with compound **e16**.

As shown in Figure 9A, there were no significant differences in body weight between the **e16**-treated and control groups. Similarly, Figure 9B shows no significant changes in the organ indices of the major organs between the groups. Biochemical analysis revealed a slight, but not statistically significant, increase in GPT levels in the **e16**-treated group (Figure 9C). Furthermore, HE staining of the major organs (heart, liver, spleen, lungs, and kidneys) showed no significant histopathological differences between the **e16**-treated and control groups (Figure 9D). These results suggest that compound **e16** has a favorable safety profile, providing experimental support for its potential clinical use.

## 4 Discussion

In this study, a series of 2H-1,4-benzoxazin-3(4H)-one derivatives were synthesized. Screening for anti-inflammatory activity revealed that compounds **e2**, **e16**, and **e20** demonstrated the best anti-inflammatory effects without significant cytotoxicity. It was found that these compounds act on Nrf2-related sites, preventing Nrf2 degradation by KEAP1. By activating the Nrf2-HO-1 signaling pathway, they reduced LPS-induced ROS levels, inhibited the expression of inflammation-related enzymes iNOS and COX-2, and decreased the production of pro-inflammatory factors such as NO, IL-1β, IL-6, and TNF-α.

Microglia-mediated neuroinflammation contributes to the development of neurodegenerative diseases. Microglia produce large amounts of inflammatory factors that disrupt neuronal function and promote neuronal death (Gao et al., 2023; Zhang et al., 2023; Cintia et al., 2024) Numerous studies have shown that intervening in microglial inflammation effectively alleviates neurodegenerative diseases (Dong et al., 2019; Sun et al., 2021). For example, rutin reduces glial proliferation and neuroinflammation in the brains of Alzheimer’s disease (AD) mice by downregulating the NF-κB pathway, inhibiting microglial phagocytosis of synapses, reducing synaptic loss, and improving cognitive performance. Dimethyl fumarate suppresses the expression of STAT3/C3 and C3 receptor in astrocytes and isolated microglia of App-KI mice, thereby inhibiting astrocyte-microglial interactions, alleviating neuroinflammation, and improving cognitive decline and behavior in AD.

Our study found that 2H-1,4-benzoxazin-3(4H)-one derivatives **e2**, **e16**, and **e20** significantly improve microglial inflammation and thus hold promise for developing therapeutics for neurodegenerative diseases.

The role of microglia in neurodegenerative diseases, toxicology, and immunology represents a rapidly expanding area of biomedical research, requiring a large number of animal experiments. Using microglia-like cell lines can accelerate research while reducing the need for continuous cell preparation and animal studies, provided the cell lines faithfully replicate *in vivo* conditions or primary microglial cells (PM). The immortalized mouse microglial cell line BV-2 is often used as a substitute for PM. Studies have reassessed the advantages and limitations of BV-2 cells and compared their response to lipopolysaccharide (LPS) with that of microglia *in vitro* and *in vivo*. Transcriptomic (480 genes) and proteomic analyses following LPS stimulation revealed that the response patterns of BV-2 cells are similar to those of PM. BV-2 cells exhibit normal regulatory and functional responses to IFN-gamma and can stimulate other glial cells. Therefore, BV-2 cells appear to be an effective substitute for PM in many experimental setups, replicating *in vivo* microglial inflammatory responses, including characteristic gene expression, secretion of inflammatory factors, cell migration, and studies of complex intercellular interactions (Kristensen et al., 2009). Due to their accessibility, ease of manipulation, and short experimental cycles, BV-2 cells are widely used as a cell model for studying microglial inflammation (Wang et al., 2021; Wang et al., 2024).

In this study, we used BV-2 microglial cells to establish an *in vitro* neuroinflammation screening model. We identified the regulatory effects of compounds **e2**, **e16**, and **e20** on microglial inflammation. These compounds significantly inhibited the expression of inflammation-related enzymes iNOS and COX-2, reduced the production of pro-inflammatory factors such as NO, IL-1β, IL-6, and TNF-α induced by LPS, and laid the foundation for further *in vivo* experiments on anti-neuroinflammation.

The Nrf2-HO-1 signaling pathway plays a crucial role in cellular oxidative stress responses and is essential in neuroinflammation. Nrf2 (nuclear factor erythroid 2-related factor 2) is a transcription factor that typically binds to Keap1 (Kelch-like ECH-associated protein 1) in the cytoplasm, maintaining Nrf2 in a low-activity state. Upon activation of the signaling pathway, Nrf2 dissociates from Keap1, translocates into the nucleus, and activates the expression of downstream genes such as HO-1, exerting antioxidant, anti-apoptotic, and anti-inflammatory effects (Li et al., 2021). Therefore, activating the Nrf2 signaling pathway can help alleviate neuroinflammation.

For instance, peppermint leaf extract induces CREB/Nrf2/HO-1-related antioxidant signaling in microglial cells, suppressing the production of pro-inflammatory enzymes and mediators such as NO, iNOS, COX-2, TNF-α, and IL-6 following LPS stimulation (Park et al., 2022). Similarly, S. alopecuroides extract alleviates the release of NO, PGE2, TNF-α, IL-6, and IL-1β induced by LPS by promoting Nrf2 nuclear translocation and HO-1 expression (Yang et al., 2016).

In this study, molecular docking analysis revealed potential binding sites of compounds **e2**, **e16**, and **e20** with Nrf2. The compounds were shown to interfere with the interaction between Keap1 and Nrf2, increasing Nrf2 protein levels, activating the downstream HO-1 antioxidant protein expression, reducing LPS-induced intracellular ROS accumulation, and alleviating neuroinflammation.

## 5 Conclusion

In this study, a series of 2H-1,4-Benzoxazin-3(4H)-one derivatives were synthesized by introducing a 1,2,3-triazole group. Screening for anti-inflammatory activity in microglial cells revealed that compounds e2, e16, and e20 exhibited the best anti-inflammatory effects without significant cytotoxicity. Consequently, these compounds were selected for further investigation of their anti-inflammatory effects and mechanisms in BV-2 microglial cells. It was found that e2, e16, and e20 not only reduced LPS-induced NO production but also significantly decreased the transcription levels of the pro-inflammatory factors IL-1β, IL-6, and TNF-α. Additionally, the transcription and protein levels of upstream inflammation-related enzymes iNOS and COX-2 were examined, revealing that these compounds significantly lowered transcription and protein levels of iNOS and COX-2 in LPS-induced BV-2 cells. To further explore the mechanisms underlying the anti-inflammatory effects of 2H-1,4-Benzoxazin-3(4H)-one derivatives in microglial cells, intracellular ROS levels and the Nrf2-HO-1 signaling pathway were investigated. The results showed that the 2H-1,4-Benzoxazin-3(4H)-one derivatives significantly activated the intracellular Nrf2-HO-1 signaling pathway, reduced LPS-induced ROS levels, and alleviated the inflammatory state of microglial cells. By molecular docking, it can be intuitively observed that compounds e2, e16, and e20 can act on Nrf2-related sites, preventing the degradation of Nrf2 by KEAP1. Additionally, acute toxicity tests in mice revealed that compound e16 exhibited good safety.

## 6 Experimental

### 6.1 Materials and chemistry

2H-1,4-Benzoxazin-3(4H)-one linked 1,2,3-triazole derivatives were synthesized in-house. Dulbecco’s modified Eagle medium (DMEM) was obtained from LifeTech (Grand Island, NY, United States). Fetal bovine serum (FBS) was purchased from Gibco (Grand Island, NY, United States). Griess reagent, β-actin antibody and Protease Inhibitor Cocktail were obtained from Sigma-Aldrich (St. Louis, Missouri, United States). Reactive Oxygen Species (ROS) Assay Kit was obtained from Beyotime Biotechnology (Shanghai, China). COX-2 antibody was purchased from Cell Signaling Technology (Beverly, MA, United States). iNOS antibody was purchased from BD Biosciences (San Jose, CA, United States). Nrf2 antibody and HO-1 antibody were purchased from Proteintech (Wuhan, China).

#### 6.1.1 General synthetic procedure for compound b (the method is applicable to the preparation of compound d)

Compound a (0.02 mol), 3-aminophenylacetylene (0.03 mol), HATU (0.03 mol), DIPEA (0.06 mol), and 250 mL of DMF were added to a 500 mL reaction flask at room temperature. The mixture was stirred under a nitrogen atmosphere for 24 h. The progress of the reaction was monitored by TLC. After 24 h, the reaction was complete, and the solution turned light brown. DMF was removed under vacuum, and the reaction mixture was extracted with dichloromethane (150 mL × 3). The combined organic layers were washed with saturated sodium chloride solution (150 mL × 2) until the pH reached 7. The organic phase was concentrated under vacuum, yielding a viscous brownish-yellow liquid. Methanol was then slowly added dropwise under ultrasonic agitation, resulting in the precipitation of a solid. The solid was filtered, washed, and dried to obtain compound b (4.6 g). ^1^H NMR(400MHz, DMSO-d_6_): 10.76 (s, 1H), 10.26 (s, 1H), 8.04 (s, 1H), 7.96 (d, J = 8.0Hz, 1H), 7.68 (d, J = 8.0Hz, 1H), 7.54–7.52 (m, 2H), 7.25–7.22 (m, 1H), 6.93 (d, J = 8.0Hz, 1H), 4.54 (s, 2H), 4.30 (s, 1H). ^13^C NMR(400MHz, DMSO-d_6_): 165.5, 164.6, 143.6, 140.0, 135.7, 134.9, 134.1, 131.0, 129.4, 128.7, 122.3, 116.4, 115.6, 108.9, 83.3, 82.0, 67.3.

#### 6.1.2 General synthetic procedure for compounds e1-e24 (the method is applicable to the preparation of compound f1-f20)

To a reaction flask, compound b (3 mmol), the substituted azide (3.6 mmol), tert-butanol (70 mL), water (70 mL), and tetrahydrofuran (70 mL) were added, followed by anhydrous copper sulfate (0.6 mmol) and sodium ascorbate (1 mmol). The mixture was stirred and refluxed at 70°C for 6 h. The progress of the reaction was monitored by TLC. Upon completion, the reaction mixture was extracted with dichloromethane (100 mL × 3), and the organic layers were combined and washed with saturated sodium chloride solution (100 mL × 2). The combined organic phase was then washed with brine (100 mL × 2), dried over sodium sulfate, and concentrated under vacuum to yield the crude product. The product was purified by recrystallization from ethyl acetate, resulting in the desired compound, which was pure enough for further characterization and anti-inflammatory studies.

The spectroscopic characterization of compounds **e1-e24** and **f1-f20** is provided as follow.

3-(1-(4-bromobenzyl)-1H-1,2,3-triazol-4-yl)-N-(3-oxo-3,4-dihydro-2H-benzo[b][1,4]oxazin-6-yl)benzamide (compound **e1**): Pure 98.9%. white solid, HR-MS(ESI): Calcd. C24H19BrN5O3 [M+H]^+^ *m/z*: 504.0671, found: 504.0657. ^1^H NMR(400MHz, DMSO-d_6_): 10.78 (s, 1H), 10.29 (s, 1H), 8.72 (s, 1H), 8.38 (s, 1H), 8.04 (d, J = 8.0Hz, 1H), 7.88 (d, J = 8.0Hz, 1H), 7.62–7.53 (m, 4H), 7.34 (d, J = 8.0Hz, 2H), 7.26 (d, J = 12.0Hz, 1H), 6.93 (d, J = 12.0Hz, 1H), 5.67 (s, 2H), 4.55 (s, 2H). ^13^C NMR(100MHz, DMSO-d_6_): 165.5, 146.6, 139.9, 136.1, 135.7, 134.3, 132.2, 131.2, 130.7, 129.4, 128.5, 127.6, 127.5, 124.8, 122.5, 121.9, 116.4, 115.6, 109.0, 67.3, 52.8.

3-(1-(3,5-dimethylbenzyl)-1H-1,2,3-triazol-4-yl)-N-(3-oxo-3,4-dihydro-2H-benzo[b][1,4]oxazin-6-yl)benzamide (compound **e2)**: Pure 99.3%. white solid, HR-MS(ESI): Calcd. C26H24N5O3 [M+H]^+^ *m/z*: 454.1879, found: 454.1964. ^1^H NMR(400MHz, DMSO-d_6_): 10.78 (s, 1H), 10.29 (s, 1H), 8.69 (s, 1H), 8.39 (s, 1H), 8.05 (d, J = 8.0Hz, 1H), 7.88 (d, J = 8.0Hz, 1H), 7.59–7.54 (m, 2H), 7.26 (d, J = 12.0Hz, 1H), 6.98–6.92 (m, 4H), 5.58 (s, 2H), 4.55 (s, 2H), 2.26 (s, 6H). ^13^C NMR(100MHz, DMSO-d_6_): 165.5, 146.5, 139.9, 138.4, 136.1, 134.3, 131.3, 130.0, 129.4, 128.4, 127.6, 127.5, 126.1, 124.8, 122.4, 116.4, 115.6, 109.0, 67.3, 53.6, 21.3.

N-(3-oxo-3,4-dihydro-2H-benzo[b][1,4]oxazin-6-yl)-3-(1-(3,4,5-trifluorobenzyl)-1H-1,2,3-triazol-4-yl)benzamide (compound **e3)**: Pure 97.6%. white solid, HR-MS(ESI): Calcd. C24H17F3N5O3 [M+H]^+^ *m/z*: 480.1283, found: 480.1282. ^1^H NMR(400MHz, DMSO-d_6_): 10.78 (s, 1H), 10.30 (s, 1H), 8.74 (s, 1H), 8.38 (s, 1H), 8.04 (d, J = 8.0Hz, 1H), 7.89 (d, J = 12.0Hz, 1H), 7.62–7.58 (m, 1H), 7.54 (s, 1H), 7.43–7.39 (m, 2H), 7.27–7.24 (m, 1H), 6.94 (d, J = 8.0Hz, 1H), 5.58 (s, 2H), 4.55 (s, 2H). ^13^C NMR(100MHz, DMSO-d_6_): 165.5, 146.6, 139.9, 136.2, 134.2, 131.1, 129.4, 128.5, 127.6, 124.9, 122.6, 113.8, 113.7, 113.6, 113.6, 108.9, 67.3, 60.2, 52.1, 21.2, 14.5.

3-(1-(2-cyano-5-fluorobenzyl)-1H-1,2,3-triazol-4-yl)-N-(3-oxo-3,4-dihydro-2H-benzo[b][1,4]oxazin-6-yl)benzamide (compound **e4)**: Pure 97.1%. white solid, HR-MS(ESI): Calcd. C25H18FN6O3 [M+H]^+^ *m/z*: 469.1424, found: 469.1412. ^1^H NMR(400MHz, DMSO-d_6_): 10.78 (s, 1H), 10.31 (s, 1H), 8.77 (s, 1H), 8.39 (s, 1H), 8.07–8.05 (m, 2H), 7.90 (d, J = 12.0Hz, 1H), 7.63–7.59 (m, 1H), 7.5–7.41 (m, 3H), 7.27–7.24 (m, 1H), 6.94 (d, J = 8.0Hz, 1H), 5.91 (s, 2H), 4.55 (s, 2H). ^13^C NMR(100MHz, DMSO-d_6_): 165.5, 146.5, 142.4, 139.9, 137.0, 136.9, 136.2, 134.3, 131.1, 129.5, 128.5, 127.6, 124.9, 123.0, 118.0, 117.7, 117.4, 117.2, 116.7, 116.4, 115.6, 109.0, 108.4, 67.3, 51.5.

3-(1-(2-chloro-6-fluorobenzyl)-1H-1,2,3-triazol-4-yl)-N-(3-oxo-3,4-dihydro-2H-benzo[b][1,4]oxazin-6-yl)benzamide (compound **e5)**: Pure 98.3%. white solid, HR-MS(ESI): Calcd. C24H18ClFN5O3 [M+H]^+^ *m/z*: 478.1082, found: 478.1063. ^1^H NMR(400MHz, DMSO-d_6_): 10.78 (s, 1H), 10.28 (s, 1H), 8.69 (s, 1H), 8.38 (s, 1H), 8.06 (d, J = 8.0Hz, 1H), 7.87 (d, J = 8.0Hz, 1H), 7.60–7.52 (m, 3H), 7.46–7.35 (m, 2H), 7.27–7.24 (m, 1H), 6.94 (d, J = 12.0Hz, 1H), 5.79 (s, 2H), 4.55 (s, 2H). ^13^C NMR(100MHz, DMSO-d_6_): 165.5, 146.2, 139.9, 136.2, 135.5, 134.3, 132.4, 132.3, 131.1, 129.4, 128.5, 127.5, 126.3, 124.9, 122.6, 116.4, 115.6, 115.3, 109.0, 67.3, 45.2.

3-(1-(3-chloro-4-fluorobenzyl)-1H-1,2,3-triazol-4-yl)-N-(3-oxo-3,4-dihydro-2H-benzo[b][1,4]oxazin-6-yl)benzamide (compound **e6)**: Pure 97.7%. white solid, HR-MS(ESI): Calcd. C24H18ClFN5O3 [M+H]^+^ *m/z*: 478.1082, found: 478.1062. ^1^H NMR(400MHz, DMSO-d_6_): 10.78 (s, 1H), 10.30 (s, 1H), 8.75 (s, 1H), 8.38 (s, 1H), 8.04 (d, J = 12.0Hz, 1H), 7.89 (d, J = 8.0Hz, 1H), 7.69–7.67 (m, 1H), 7.60 (t, J_1_ = 8.0Hz, J_2_ = 8.0Hz, 1H), 7.54 (s, 1H), 7.46–7.42 (m, 2H), 7.25 (dd, J_1_ = 4.0Hz, J_2_ = 4.0Hz, 1H), 6.94 (d, J = 8.0Hz, 1H), 5.69 (s, 2H), 4.55 (s, 2H). ^13^C NMR(100MHz, DMSO-d_6_): 165.5, 146.6, 139.9, 136.1, 134.3, 130.9, 129.6, 129.5, 129.4, 128.5, 127.5, 124.9, 122.5, 120.2, 120.1, 117.9, 117.7, 116.4, 115.6, 109.0, 67.3, 52.2.

3-(1-(3-chlorobenzyl)-1H-1,2,3-triazol-4-yl)-N-(3-oxo-3,4-dihydro-2H-benzo[b][1,4]oxazin-6-yl)benzamide (compound e7): Pure 96.5%. white solid, HR-MS(ESI): Calcd. C24H19ClN5O3 [M+H]^+^ *m/z*: 460.1176, found: 460.1163. ^1^H NMR(400MHz, DMSO-d_6_): 10.78 (s, 1H), 10.30 (s, 1H), 8.76 (s, 1H), 8.39 (s, 1H), 8.05 (d, J = 12.0Hz, 1H), 7.89 (d, J = 8.0Hz, 1H), 7.60 (t, J = 8.0Hz, 1H), 7.54 (s, 1H), 7.48–7.43 (m, 3H), 7.35–7.32 (m, 1H), 7.26 (dd, J_1_ = 4.0Hz, J_2_ = 4.0Hz, 1H), 6.94 (d, J = 8.0Hz, 1H), 5.71 (s, 2H), 4.55 (s, 2H). ^13^C NMR(100MHz, DMSO-d_6_): 165.5, 146.6, 139.9, 138.7, 136.1, 134.3, 133.8, 131.2, 129.4, 128.7, 128.5, 128.3, 127.6, 127.1, 124.9, 122.6, 116.4, 115.6, 109.0, 67.3, 52.8.

3-(1-(5-chloro-2-fluorobenzyl)-1H-1,2,3-triazol-4-yl)-N-(3-oxo-3,4-dihydro-2H-benzo[b][1,4]oxazin-6-yl)benzamide (compound **e8)**: Pure 97.9%. white solid, HR-MS(ESI): Calcd. C24H18ClFN5O3 [M+H]^+^ *m/z*: 478.1082, found: 478.1063. ^1^H NMR(400MHz, DMSO-d_6_): 10.78 (s, 1H), 10.30 (s, 1H), 8.74 (s, 1H), 8.39 (s, 1H), 8.07 (s, 1H), 7.88 (s, 1H), 7.61–7.25 (m, 6H), 6.94 (d, J = 8.0Hz, 1H), 5.74 (s, 2H), 4.55 (s, 2H). ^13^C NMR(100MHz, DMSO-d_6_): 165.5, 160.7, 158.2, 146.5, 139.9, 138.5, 136.2, 134.3, 131.1, 131.0, 129.4, 128.5, 127.6, 125.2, 125.1, 124.9, 122.6, 118.3, 118.1, 116.4, 115.6, 109.0, 67.3, 47.3.

3-(1-(4-fluorobenzyl)-1H-1,2,3-triazol-4-yl)-N-(3-oxo-3,4-dihydro-2H-benzo[b][1,4]oxazin-6-yl)benzamide (compound **e9)**: Pure 95.2%. white solid, HR-MS(ESI): Calcd. C24H19FN5O3 [M+H]^+^ *m/z*: 444.1472, found: 444.1479. ^1^H NMR(400MHz, DMSO-d_6_): 10.80 (s, 1H), 10.31 (s, 1H), 8.73 (s, 1H), 8.38 (s, 1H), 8.06–8.04 (m, 1H), 7.88 (d, J = 8.0Hz, 1H), 7.62–7.44 (m, 4H), 7.27–7.24 (m, 2H), 6.94 (d, J = 8.0Hz, 1H), 5.68 (s, 2H), 4.56 (s, 2H). ^13^C NMR(100MHz, DMSO-d_6_): 165.5, 163.6, 161.2, 146.6, 139.8, 136.1, 135.8, 134.2, 132.6, 131.2, 129.5, 128.4, 127.5, 124.8, 122.4, 116.4, 116.2, 116.0, 115.6, 108.9, 67.3, 52.8.

3-(1-(2-fluorobenzyl)-1H-1,2,3-triazol-4-yl)-N-(3-oxo-3,4-dihydro-2H-benzo[b][1,4]oxazin-6-yl)benzamide (compound **e10)**: Pure 96.7%. white solid, HR-MS(ESI): Calcd. C24H19FN5O3 [M+H]^+^ *m/z*: 444.1472, found: 444.1465. ^1^H NMR(400MHz, DMSO-d_6_): 10.78 (s, 1H), 10.29 (s, 1H), 8.71 (s, 1H), 8.39 (s, 1H), 8.04 (d, J = 8.0Hz, 1H), 7.88 (d, J = 8.0Hz, 1H), 7.61–7.26 (m, 7H), 6.94 (d, J = 8.0Hz, 1H), 5.74 (s, 2H), 4.55 (s, 1H). ^13^C NMR(100MHz, DMSO-d_6_): 165.5, 146.5, 139.9, 136.1, 134.3, 131.2, 129.4, 128.5, 127.6, 125.4, 124.8, 123.2, 123.0, 122.5, 116.4, 116.2, 116.0, 115.6, 109.0, 67.3, 47.6.

3-(1-(3,5-dibromobenzyl)-1H-1,2,3-triazol-4-yl)-N-(3-oxo-3,4-dihydro-2H-benzo[b][1,4]oxazin-6-yl)benzamide (compound **e11)**: Pure 98.4%. white solid, HR-MS(ESI): Calcd. C24H18Br2N5O3 [M+H]^+^ *m/z*: 581.9776, found: 581.9769. ^1^H NMR(400MHz, DMSO-d_6_): 10.78 (s, 1H), 10.30 (s, 1H), 8.78 (s, 1H), 8.39 (s, 1H), 8.06 (d, J = 8.0Hz, 1H), 7.91–7.82 (m, 2H), 7.64–7.59 (m, 3H), 7.55 (d, J = 4.0Hz, 1H), 7.27–7.25 (m, 1H), 6.94 (d, J = 8.0Hz, 1H), 5.71 (s, 2H), 4.55 (s, 1H). ^13^C NMR(100MHz, DMSO-d_6_): 165.5, 146.7, 140.7, 139.9, 136.2, 134.3, 133.7, 130.6, 129.5, 128.5, 127.6, 124.9, 123.2, 122.8, 116.4, 115.6, 109.0, 67.3, 52.4, 52.0, 49.0.

3-(1-(4-bromo-2-fluorobenzyl)-1H-1,2,3-triazol-4-yl)-N-(3-oxo-3,4-dihydro-2H-benzo[b][1,4]oxazin-6-yl)benzamide (compound **e12)**: Pure 95.7%. white solid, HR-MS(ESI): Calcd. C24H18BrFN5O3 [M+H]^+^ *m/z*: 522.0577, found: 522.0571. ^1^H NMR(400MHz, DMSO-d_6_): 10.77 (s, 1H), 10.29 (s, 1H), 8.70 (s, 1H), 8.38 (s, 1H), 8.05 (d, J = 8.0Hz, 1H), 7.88 (d, J = 8.0Hz, 1H), 7.66–7.37 (m, 5H), 7.25 (d, J = 8.0Hz, 1H), 6.93 (d, J = 8.0Hz, 1H), 5.72 (s, 2H), 4.55 (s, 1H). ^13^C NMR(100MHz, DMSO-d_6_): 165.5, 146.5, 139.9, 136.1, 134.2, 132.9, 132.8, 131.1, 129.4, 128.6, 128.5, 128.5, 127.5, 124.8, 122.8, 122.6, 119.7, 119.5, 116.4, 115.6, 109.0, 67.3, 47.2.

N-(3-oxo-3,4-dihydro-2H-benzo[b][1,4]oxazin-6-yl)-3-(1-(3-(trifluoromethyl)benzyl)-1H-1,2,3-triazol-4-yl)benzamide (compound **e13)**: Pure 94.8%. white solid, HR-MS(ESI): Calcd. C25H19F3N5O3 [M+H]^+^ *m/z*: 494.1440, found: 494.1429. ^1^H NMR(400MHz, DMSO-d_6_): 10.78 (s, 1H), 10.30 (s, 1H), 8.79 (s, 1H), 8.39 (s, 1H), 8.06–7.55 (m, 8H), 7.26 (d, J = 8.0Hz, 1H), 6.94 (d, J = 8.0Hz, 1H), 5.82 (s, 2H), 4.55 (s, 2H). ^13^C NMR(100MHz, DMSO-d_6_): 165.5, 139.9, 137.7, 136.1, 134.3, 132.6, 131.2, 130.5, 129.5, 128.5, 127.6, 125.5, 125.4, 125.1, 124.9, 122.7, 116.4, 115.6, 109.0, 67.3, 52.8.

3-(1-(2,6-dichlorobenzyl)-1H-1,2,3-triazol-4-yl)-N-(3-oxo-3,4-dihydro-2H-benzo[b][1,4]oxazin-6-yl)benzamide (compound e14): Pure 97.7%. white solid, HR-MS(ESI): Calcd. C24H18Cl2N5O3 [M+H]^+^ *m/z*: 494.0787, found: 494.0780. ^1^H NMR(400MHz, DMSO-d_6_): 10.78 (s, 1H), 10.28 (s, 1H), 8.65 (s, 1H), 8.38 (s, 1H), 8.06 (d, J = 8.0Hz, 1H), 7.86 (d, J = 12.0Hz, 1H), 7.62–7.48 (m, 5H), 7.25 (d, J = 8.0Hz, 1H), 6.93 (d, J = 8.0Hz, 1H), 5.88 (s, 2H), 4.55 (s, 2H). ^13^C NMR(100MHz, DMSO-d_6_): 165.6, 165.5, 146.1, 139.9, 136.5, 136.2, 134.3, 132.1, 131.1, 130.6, 129.5, 129.4, 128.5, 127.5, 127.5, 124.9, 122.5, 116.3, 115.6, 108.9, 67.3, 49.3.

3-(1-(2-cyanobenzyl)-1H-1,2,3-triazol-4-yl)-N-(3-oxo-3,4-dihydro-2H-benzo[b][1,4]oxazin-6-yl)benzamide (compound **e15)**: Pure 96.8%. white solid, HR-MS(ESI): Calcd. C25H19N6O3 [M+H]^+^ *m/z*: 451.1519, found: 451.1507. ^1^H NMR(400MHz, DMSO-d_6_): 10.79 (s, 1H), 10.31 (s, 1H), 8.77 (s, 1H), 8.40 (s, 1H), 8.07 (d, J = 8.0Hz, 1H), 7.92 (dd, J_1_ = 8.0Hz, J_2_ = 8.0Hz, 2H), 7.77 (t, J = 8.0Hz, 1H), 7.63–7.47 (m, 4H), 7.28–7.25 (m, 1H), 6.94 (d, J = 8.0Hz, 1H), 5.91 (s, 2H), 4.56 (s, 2H). ^13^C NMR(100MHz, DMSO-d_6_): 165.5, 146.5, 139.9, 139.0, 136.2, 134.3, 134.3, 133.9, 131.1, 130.0, 129.7, 129.5, 128.5, 127.6, 124.9, 122.9, 117.4, 116.4, 115.6, 111.7, 109.0, 67.3, 51.8.

N-(3-oxo-3,4-dihydro-2H-benzo[b][1,4]oxazin-6-yl)-3-(1-(4-(trifluoromethyl)benzyl)-1H-1,2,3-triazol-4-yl)benzamide (compound **e16)**: Pure 94.2%. white solid, HR-MS(ESI): Calcd. C25H19F3N5O3 [M+H]^+^ *m/z*: 494.1440, found: 494.1435. ^1^H NMR(400MHz, DMSO-d_6_): 10.78 (s, 1H), 10.30 (s, 1H), 8.78 (s, 1H), 8.39 (s, 1H), 8.06 (d, J = 12.0Hz, 1H), 7.91–7.88 (m, 1H), 7.78 (d, J = 8.0Hz, 2H), 7.60–7.54 (m, 4H), 7.25 (dd, J_1_ = 4.0Hz, J_2_ = 4.0Hz, 1H), 6.94 (d, J = 8.0Hz, 1H), 5.82 (s, 2H), 4.55 (s, 2H). ^13^C NMR(100MHz, DMSO-d_6_): 165.5, 146.7, 141.0, 139.9, 136.1, 134.3, 131.2, 129.5, 129.3, 129.1, 129.0, 128.5, 127.6, 126.2, 126.2, 124.9, 122.8, 116.4, 115.6, 109.0, 67.3, 52.9.

3-(1-(2-bromobenzyl)-1H-1,2,3-triazol-4-yl)-N-(3-oxo-3,4-dihydro-2H-benzo[b][1,4]oxazin-6-yl)benzamide (compound **e17)**: Pure 99.0%. white solid, HR-MS(ESI): Calcd. C24H19BrN5O3 [M+H]^+^ *m/z*: 504.0671, found: 504.0666. ^1^H NMR(400MHz, DMSO-d_6_): 10.80 (s, 1H), 10.31 (s, 1H), 8.70 (s, 1H), 8.40 (s, 1H), 8.07 (d, J = 8.0Hz, 1H), 7.89 (d, J = 8.0Hz, 1H), 7.72 (d, J = 8.0Hz, 2H), 7.62–7.24 (m, 6H), 6.94 (d, J = 8.0Hz, 1H), 5.77 (s, 2H), 4.55 (s, 2H). ^13^C NMR(100MHz, DMSO-d_6_): 165.5, 146.4, 139.9, 136.1, 135.1, 134.3, 133.4, 131.2, 131.1, 131.0, 129.5, 128.8, 128.5, 127.5, 124.8, 123.4, 122.8, 122.5, 116.4, 115.6, 108.9, 67.3, 53.7.

3-(1-(3-fluorobenzyl)-1H-1,2,3-triazol-4-yl)-N-(3-oxo-3,4-dihydro-2H-benzo[b][1,4]oxazin-6-yl)benzamide (compound **e18)**: Pure 98.7%. white solid, HR-MS(ESI): Calcd. C24H19FN5O3 [M+H]^+^ *m/z*: 444.1472, found: 444.1473. ^1^H NMR(400MHz, DMSO-d_6_): 10.77 (s, 1H), 10.29 (s, 1H), 8.75 (s, 1H), 8.38 (s, 1H), 8.05 (d, J = 12.0Hz, 1H), 7.88 (d, J = 8.0Hz, 1H), 7.59 (t, J = 8.0Hz, 1H), 7.54 (d, J = 4.0Hz, 1H), 7.48–7.43 (m, 1H), 7.26–7.17 (m, 4H), 6.93 (d, J = 8.0Hz, 1H), 5.71 (s, 2H), 4.54 (s, 2H). ^13^C NMR(100MHz, DMSO-d_6_): 165.5, 146.6, 139.9, 139.0, 136.1, 134.3, 131.4, 131.3, 131.2, 129.4, 128.5, 127.6, 127.5, 124.8, 124.5, 124.5, 122.6, 116.4, 115.6, 115.4, 115.2, 109.0, 67.3, 52.9.

3-(1-(4-iodobenzyl)-1H-1,2,3-triazol-4-yl)-N-(3-oxo-3,4-dihydro-2H-benzo[b][1,4]oxazin-6-yl)benzamide (compound **e19)**: Pure 97.6%. white solid, HR-MS(ESI): Calcd. C24H19IN5O3 [M+H]^+^ *m/z*: 552.0533, found: 552.0547. ^1^H NMR(400MHz, DMSO-d_6_): 10.77 (s, 1H), 10.28 (s, 1H), 8.71 (s, 1H), 8.37 (s, 1H), 8.04 (d, J = 12.0Hz, 1H), 7.88 (d, J = 8.0Hz, 1H), 7.11 (d, J = 8.0Hz, 2H), 7.61–7.53 (m, 2H), 7.26–7.17 (m, 3H), 6.93 (d, J = 8.0Hz, 1H), 5.65 (s, 2H), 4.54 (s, 2H). ^13^C NMR(100MHz, DMSO-d_6_): 165.5, 165.5, 146.6, 139.9, 138.0, 136.1, 134.3, 131.2, 130.7, 129.5, 128.4, 127.5, 127.5, 124.8, 122.5, 116.4, 115.6, 108.9, 95.0, 67.3, 53.0.

3-(1-(2-nitrobenzyl)-1H-1,2,3-triazol-4-yl)-N-(3-oxo-3,4-dihydro-2H-benzo[b][1,4]oxazin-6-yl)benzamide (compound **e20)**: Pure 95.5%. white solid, HR-MS(ESI): Calcd. C24H19N6O5 [M+H]^+^ *m/z*: 471.1417, found: 471.1409. ^1^H NMR(400MHz, DMSO-d_6_): 10.80 (s, 1H), 10.32 (s, 1H), 8.81 (s, 1H), 8.39 (s, 1H), 8.30–8.22 (m, 2H), 8.05 (d, J = 8.0Hz, 1H), 7.87 (dd, J_1_= 8.0Hz, J2= 8.0Hz, 2H), 7.72 (t, J = 8.0Hz, 1H), 7.61 (t, J = 4.0Hz, 1H), 7.55 (d, J = 4.0Hz, 1H), 7.27–7.24 (m, 1H), 6.94 (d, J = 8.0Hz, 1H), 5.88 (s, 2H), 4.55 (s, 2H). ^13^C NMR(100MHz, DMSO-d_6_): 165.5, 165.5, 148.4, 146.7, 139.9, 138.4, 136.1, 135.2, 134.2, 131.1, 130.9, 129.5, 128.5, 127.5, 124.8, 123.7, 123.3, 122.8, 116.4, 115.6, 108.9, 67.3, 52.5.

3-(1-(4-chlorobenzyl)-1H-1,2,3-triazol-4-yl)-N-(3-oxo-3,4-dihydro-2H-benzo[b][1,4]oxazin-6-yl)benzamide (compound **e21)**: Pure 97.1%. white solid, HR-MS(ESI): Calcd. C24H19ClN5O3 [M+H]^+^ *m/z*: 460.1176, found: 460.1191. ^1^H NMR(400MHz, DMSO-d_6_): 10.80 (s, 1H), 10.31 (s, 1H), 9.42 (s, 1H), 8.74 (s, 1H), 8.38 (s, 1H), 8.04 (d, J = 8.0Hz, 1H), 7.88 (d, J = 8.0Hz, 1H), 7.59 (t, J = 8.0Hz, 1H), 7.54 (d, J = 4.0Hz, 1H), 7.40 (d, J = 8.0Hz, 2H), 7.26–7.24 (m, 1H), 6.94 (d, J = 8.0Hz, 1H), 5.69 (s, 2H),4.55 (s, 2H). ^13^C NMR(100MHz, DMSO-d_6_): 165.5, 146.6, 144.3, 139.9, 135.3, 134.2, 132.2, 131.2, 130.4, 129.5, 129.3, 128.4, 127.5, 127.5, 122.4, 116.4, 115.6, 108.9, 67.3, 52.7.

N-(3-oxo-3,4-dihydro-2H-benzo[b][1,4]oxazin-6-yl)-3-(1-(2-(trifluoromethyl)benzyl)-1H-1,2,3-triazol-4-yl)benzamide (compound **e22)**: Pure 96.7%. white solid, HR-MS(ESI): Calcd. C25H19F3N5O3 [M+H]^+^ *m/z*: 494.1440, found: 494.1447. ^1^H NMR(400MHz, DMSO-d_6_): 10.80 (s, 1H), 10.32 (s, 1H), 8.73 (s, 1H), 8.41 (s, 1H), 8.07 (d, J = 8.0Hz, 1H), 7.90–7.83 (m, 2H), 7.72 (t, J = 8.0Hz, 1H), 7.63–7.54 (m, 3H), 7.30–7.24 (m, 2H), 6.94 (d, J = 8.0Hz, 1H), 5.88 (s, 2H), 4.55 (s, 2H). ^13^C NMR(100MHz, DMSO-d_6_):165.5, 146.5, 139.9, 136.1, 134.2, 133.9, 133.7, 131.1, 130.8, 129.4, 128.5, 127.5, 126.7, 124.9, 123.0, 116.4, 115.6, 108.9, 67.3, 50.3.

3-(1-benzyl-1H-1,2,3-triazol-4-yl)-N-(3-oxo-3,4-dihydro-2H-benzo[b][1,4]oxazin-6-yl)benzamide (compound **e23)**: Pure 97.6%. white solid, HR-MS(ESI): Calcd. C24H20N5O3 [M+H]^+^ *m/z*: 426.1566, found: 426.1567. ^1^H NMR(400MHz, DMSO-d_6_): 10.79 (s, 1H), 10.30 (s, 1H), 8.74 (s, 1H), 8.38 (s, 1H), 8.05 (d, J = 8.0Hz, 1H), 7.88 (d, J = 8.0Hz, 1H), 7.61–7.53 (m, 2H), 7.43–7.34 (m, 5H), 7.26–7.23 (m, 1H), 6.93 (d, J = 12.0Hz, 1H), 5.68 (s, 2H), 4.55 (s, 2H). ^13^C NMR(100MHz, DMSO-d_6_): 165.5, 146.6, 136.4, 136.1, 134.2, 133.0, 131.2, 129.3, 128.7, 128.4, 128.4, 127.5, 127.5, 122.5, 116.4, 115.6, 112.1, 108.9, 108.7, 67.3, 53.5.

3-(1-(2-chloro-5-fluorobenzyl)-1H-1,2,3-triazol-4-yl)-N-(3-oxo-3,4-dihydro-2H-benzo[b][1,4]oxazin-6-yl)benzamide (compound **e24)**: Pure 97.6%. white solid, HR-MS(ESI): Calcd. C24H18ClFN5O3 [M+H]^+^ *m/z*: 478.1082, found: 478.1075. ^1^H NMR(400MHz, DMSO-d_6_): 10.81 (s, 1H), 10.33 (s, 1H), 8.74 (s, 1H), 8.41 (s, 1H), 8.08 (d, J = 8.0Hz, 1H), 7.90 (d, J = 8.0Hz, 2H), 7.63–7.55 (m, 3H), 7.36–7.26 (m, 3H), 6.95 (d, J = 8.0Hz, 1H), 5.79 (s, 2H), 4.56 (s, 2H).^13^C NMR(100MHz, DMSO-d_6_): 165.5, 162.5, 160.0, 146.4, 139.9, 136.2, 135.6, 135.5, 134.3, 132.0, 131.9, 131.1, 129.4, 128.7, 128.5, 127.6, 124.9, 122.8, 118.4, 118.1, 117.9, 117.7, 116.4, 115.6, 108.9, 67.3, 51.1.

3-(1-(2,6-dichlorobenzyl)-1H-1,2,3-triazol-4-yl)-N-(3-oxo-3,4-dihydro-2H-benzo[b][1,4]oxazin-7-yl)benzamide (compound **f1)**: Pure 97.1%. white solid, HR-MS(ESI): Calcd. C24H18Cl2N5O3 [M+H]^+^ *m/z*: 494.0787, found: 494.0775. ^1^H NMR(400MHz, DMSO-d_6_): 10.67 (s, 1H), 10.27 (s, 1H), 8.66 (s, 1H), 8.38 (s, 1H), 8.07 (d, J = 8.0Hz, 1H), 7.87 (d, J = 8.0Hz, 1H), 7.62–7.56 (m, 3H), 7.52–7.45 (m, 2H), 7.38–7.35 (m, 1H), 6.88 (d, J = 8.0Hz, 1H), 5.88 (s, 2H), 4.57 (s, 2H). ^13^C NMR(100MHz, DMSO-d_6_): 165.6, 164.9, 146.0, 143.5, 136.5, 136.1, 135.0, 132.1, 131.2, 130.6, 129.5, 129.4, 129.3, 128.6, 127.5, 124.9, 123.6, 122.5, 116.0, 114.8, 109.1, 67.3, 49.3.

3-(1-(3-chlorobenzyl)-1H-1,2,3-triazol-4-yl)-N-(3-oxo-3,4-dihydro-2H-benzo[b][1,4]oxazin-7-yl)benzamide (compound **f2)**: Pure 98.4%. white solid, HR-MS(ESI): Calcd. C24H19ClN5O3 [M+H]^+^ *m/z*: 460.1176, found: 460.1162. ^1^H NMR(400MHz, DMSO-d_6_): 10.66 (s, 1H), 10.28 (s, 1H), 8.77 (s, 1H), 8.39 (s, 1H), 8.06 (d, J = 4.0Hz, 1H), 7.88 (d, J = 8.0Hz, 1H), 7.60 (t, J = 8.0Hz, 1H), 7.50–7.48 (m, 2H), 7.44–7.33 (m, 4H), 6.88 (d, J = 8.0Hz, 1H), 5.71 (s, 2H), 4.58 (s, 2H).^13^C NMR(100MHz, DMSO-d_6_): 165.6, 164.9, 146.6, 143.5, 138.7, 136.1, 135.0, 133.8, 131.2, 129.5, 128.7, 128.5, 128.3, 127.5, 127.1, 124.8, 123.6, 122.6, 116.0, 114.8, 109.1, 67.3, 52.8, 49.0.

3-(1-(2-fluorobenzyl)-1H-1,2,3-triazol-4-yl)-N-(3-oxo-3,4-dihydro-2H-benzo[b][1,4]oxazin-7-yl)benzamide (compound **f3)**: Pure 95.9%. white solid, HR-MS(ESI): Calcd. C24H19FN5O3 [M+H]^+^ *m/z*: 444.1472, found: 444.1463. ^1^H NMR(400MHz, DMSO-d_6_): 10.67 (s, 1H), 10.27 (s, 1H), 8.72 (s, 1H), 8.39 (s, 1H), 8.06 (d, J = 4.0Hz, 1H), 7.88 (d, J = 8.0Hz, 1H), 7.59 (t, J = 8.0Hz, 1H), 7.50–7.26 (m, 6H), 6.88 (d, J = 8.0Hz, 1H), 5.74 (s, 2H), 4.58 (s, 2H).^13^C NMR(100MHz, DMSO-d_6_): 165.6, 164.9, 161.8, 159.4, 146.5, 143.5, 136.1, 135.0, 131.3, 129.5, 128.5, 127.5, 125.4, 124.8, 123.6, 123.2, 123.0, 122.6, 116.2, 116.0, 114.9, 109.1, 67.3, 47.6.

3-(1-(4-iodobenzyl)-1H-1,2,3-triazol-4-yl)-N-(3-oxo-3,4-dihydro-2H-benzo[b][1,4]oxazin-7-yl)benzamide (compound **f4)**: Pure 97.8%. white solid, HR-MS(ESI): Calcd. C24H19IN5O3 [M+H]^+^ *m/z*: 552.0533, found: 552.0515. ^1^H NMR(400MHz, DMSO-d_6_): 10.70 (s, 1H), 10.30 (s, 1H), 8.73 (s, 1H), 8.37 (s, 1H), 8.05 (d, J = 4.0Hz, 1H), 7.88 (d, J = 8.0Hz, 1H), 7.78 (d, J = 8.0Hz, 2H), 7.60 (t, J = 8.0Hz, 1H), 7.49 (s, 1H), 7.26 (dd, J_1_ = 4.0Hz, J_2_ = 4.0Hz, 1H), 7.18 (d, J = 4.0Hz, 2H), 6.87 (d, J = 8.0Hz, 1H), 5.65 (s, 2H), 4.58 (s, 2H).^13^C NMR(100MHz, DMSO-d_6_): 165.5, 165.0, 146.6, 143.5, 138.0, 136.1, 136.1, 135.0, 131.2, 130.7, 129.5, 128.5, 127.5, 124.8, 123.6, 122.5, 114.8, 109.1, 95.0, 67.2, 52.9.

3-(1-(4-bromobenzyl)-1H-1,2,3-triazol-4-yl)-N-(3-oxo-3,4-dihydro-2H-benzo[b][1,4]oxazin-7-yl)benzamide (compound **f5)**: Pure 94.8%. white solid, HR-MS(ESI): Calcd. C24H19BrN5O3 [M+H]^+^ *m/z*: 504.0671, found: 504.0665. ^1^H NMR(400MHz, DMSO-d_6_): 10.67 (s, 1H), 10.28 (s, 1H), 8.71 (s, 1H), 8.36 (s, 1H), 8.04 (d, J = 8.0Hz, 1H), 7.88 (d, J = 8.0Hz, 1H), 7.60 (d, J = 8.0Hz, 2H), 7.48 (s, 1H), 7.37–7.33 (m, 3H), 6.88 (d, J = 8.0Hz, 1H), 5.66 (s, 2H), 4.57 (s, 2H).^13^C NMR(100MHz, DMSO-d_6_): 165.6, 165.0, 146.6, 143.5, 136.0, 135.7, 134.9, 132.2, 131.2, 130.7, 129.5, 128.5, 127.5, 124.8, 123.6, 122.5, 121.9, 116.0, 114.9, 109.2, 67.2, 52.8.

3-(1-(3,5-dimethylbenzyl)-1H-1,2,3-triazol-4-yl)-N-(3-oxo-3,4-dihydro-2H-benzo[b][1,4]oxazin-7-yl)benzamide (compound **f6**): Pure 96.2%. white solid, HR-MS(ESI): Calcd. C26H24N5O3 [M+H]^+^ *m/z*: 454.1879, found: 454.1867. ^1^H NMR(400MHz, DMSO-d_6_): 10.67 (s, 1H), 10.28 (s, 1H), 8.71 (s, 1H), 8.39 (s, 1H), 8.06 (d, J = 8.0Hz, 1H), 7.88 (d, J = 8.0Hz, 1H), 7.60 (t, J = 4.0Hz, 2H), 7.50 (s, 1H), 7.38 (dd, J_1_ = 4.0Hz, J_2_ = 4.0Hz, 1H), 6.99 (s, 3H), 6.88 (d, J = 8.0Hz, 1H), 5.58 (s, 2H), 4.58 (s, 2H), 2.26 (s, 6H).^13^C NMR(100MHz, DMSO-d_6_): 165.6, 164.9, 146.5, 143.5, 138.4, 136.1, 136.1, 135.0, 131.3, 130.0, 129.5, 128.5, 127.4, 124.8, 123.6, 122.4, 116.0, 114.9, 109.1, 67.3, 53.6, 21.3.

3-(1-(2-cyanobenzyl)-1H-1,2,3-triazol-4-yl)-N-(3-oxo-3,4-dihydro-2H-benzo[b][1,4]oxazin-7-yl)benzamide (compound **f7)**: Pure 95.8%. white solid, HR-MS(ESI): Calcd. C25H19N6O3 [M+H]^+^ *m/z*: 451.1519, found: 451.1504. ^1^H NMR(400MHz, DMSO-d_6_): 10.67 (s, 1H), 10.29 (s, 1H), 8.75 (s, 1H), 8.38 (s, 1H), 8.06 (d, J = 8.0Hz, 1H), 7.94 (d, J = 8.0Hz, 1H), 7.89 (d, J = 8.0Hz, 1H), 7.76 (t, J = 8.0Hz, 1H), 7.63–7.59 (m, 2H), 7.50–7.48 (m, 2H), 7.36 (dd, J_1_ = 4.0Hz, J_2_ = 4.0Hz, 1H), 6.88 (d, J = 8.0Hz, 1H), 5.90 (s, 2H), 4.58 (s, 2H). ^13^C NMR(100MHz, DMSO-d_6_): 165.6, 165.0, 146.5, 143.5, 138.9, 136.1, 135.0, 134.3, 133.9, 131.1, 130.1, 129.8, 129.5, 128.6, 127.5, 124.9, 123.6, 122.9, 117.4, 116.0, 114.9, 111.7, 109.2, 67.2, 51.8.

N-(3-oxo-3,4-dihydro-2H-benzo[b][1,4]oxazin-7-yl)-3-(1-(3-(trifluoromethyl)benzyl)-1H-1,2,3-triazol-4-yl)benzamide (compound **f8**): Pure 97.2%. white solid, HR-MS(ESI): Calcd. C25H19F5N5O3 [M+H]^+^ *m/z*: 494.1440, found: 494.1433. ^1^H NMR(400MHz, DMSO-d_6_): 10.62 (s, 1H), 10.24 (s, 1H), 8.70 (s, 1H), 8.31 (s, 1H), 7.99 (d, J = 8.0Hz, 1H), 7.82 (d, J = 8.0Hz, 1H), 7.71–7.66 (m, 2H), 7.60–7.53 (m, 3H), 7.42 (s, 1H), 7.29 (d, J = 8.0Hz, 1H), 6.84 (d, J = 8.0Hz, 1H), 5.74 (s, 2H), 4.58 (s, 2H). ^13^C NMR(100MHz, DMSO-d_6_): 165.7, 165.1, 146.6, 143.5, 137.6, 136.0, 134.9, 132.6, 131.1, 130.5, 130.1, 129.8, 129.6, 128.6, 127.5, 125.8, 125.5, 125.5, 125.0, 125.0, 124.8, 123.6, 123.0, 122.6, 116.0, 115.0, 109.2, 67.2, 52.8.

3-(1-(3-chloro-4-fluorobenzyl)-1H-1,2,3-triazol-4-yl)-N-(3-oxo-3,4-dihydro-2H-benzo[b][1,4]oxazin-7-yl)benzamide (compound **f9**): Pure 98.0%. white solid, HR-MS(ESI): Calcd. C24H18ClFN5O3 [M+H]^+^ *m/z*: 478.1082, found: 478.1073. ^1^H NMR(400MHz, DMSO-d_6_): 10.68 (s, 1H), 10.29 (s, 1H), 8.75 (s, 1H), 8.38 (s, 1H), 8.06 (d, J = 4.0Hz, 1H), 7.89 (d, J = 8.0Hz, 1H), 7.69–7.59 (m, 2H), 7.50–7.36 (m, 4H), 6.89 (d, J = 8.0Hz, 1H), 5.70 (s, 2H), 4.58 (s, 2H). ^13^C NMR(100MHz, DMSO-d_6_): 165.6, 165.0, 158.7, 146.6, 143.5, 136.1, 135.0, 134.1, 134.1, 131.2, 131.0, 130.9, 129.7, 129.6, 129.5, 129.5, 129.5, 128.5, 127.5, 124.8, 123.6, 122.5, 117.9, 117.7, 116.0, 114.9, 109.2, 67.2, 52.6, 52.2.

3-(1-(2,6-difluorobenzyl)-1H-1,2,3-triazol-4-yl)-N-(3-oxo-3,4-dihydro-2H-benzo[b][1,4]oxazin-7-yl)benzamide (compound **f10)**: Pure 96.9%. white solid, HR-MS(ESI): Calcd. C24H18F2N5O3 [M+H]^+^ *m/z*: 462.1378, found: 462.1370. ^1^H NMR(400MHz, DMSO-d_6_): 10.66 (s, 1H), 10.26 (s, 1H), 8.71 (s, 1H), 8.38 (s, 1H), 8.06 (d, J = 4.0Hz, 1H), 7.87 (d, J = 8.0Hz, 1H), 7.61–7.49 (m, 3H), 7.38 (d, J = 4.0Hz, 1H), 7.21 (t, J = 8.0Hz, 2H), 6.88 (d, J = 8.0Hz, 1H), 5.74 (s, 2H), 4.58 (s, 2H). ^13^C NMR(100MHz, DMSO-d_6_): 165.6, 164.9, 146.4, 143.5, 136.1, 132.2, 131.1, 129.4, 128.5, 127.5, 124.9, 123.6, 122.5, 116.0, 114.9, 112.5, 112.3, 109.1, 67.3.

3-(1-(2-cyano-5-fluorobenzyl)-1H-1,2,3-triazol-4-yl)-N-(3-oxo-3,4-dihydro-2H-benzo[b][1,4]oxazin-7-yl)benzamide (compound **f11)**: Pure 96.5%. white solid, HR-MS(ESI): Calcd. C25H18FN6O3 [M+H]^+^ *m/z*: 469.1424, found: 469.1410. ^1^H NMR(400MHz, DMSO-d_6_): 10.66 (s, 1H), 10.29 (s, 1H), 8.77 (s, 1H), 8.38 (s, 1H), 8.07–8.04 (m, 1H), 7.89 (d, J = 8.0Hz, 1H), 7.61 (t, J = 8.0Hz, 1H), 7.49–7.35 (m, 4H), 6.88 (d, J = 8.0Hz, 1H), 5.90 (s, 2H), 4.58 (s, 2H). ^13^C NMR(100MHz, DMSO-d_6_): 166.2, 165.6, 164.9, 163.7, 146.5, 143.5, 142.4, 142.3, 137.0, 136.9, 136.1, 135.0, 131.1, 129.5, 128.6, 127.6, 124.9, 123.6, 123.0, 118.0, 117.7, 117.5, 117.2, 116.7, 116.0, 114.9, 109.1, 108.5, 108.4, 67.3, 51.5.

3-(1-(2-bromobenzyl)-1H-1,2,3-triazol-4-yl)-N-(3-oxo-3,4-dihydro-2H-benzo[b][1,4]oxazin-7-yl)benzamide (compound **f12**): Pure 98.2%. white solid, HR-MS(ESI): Calcd. C24H19BrN5O3 [M+H]^+^ *m/z*: 504.0671, found: 504.0666. ^1^H NMR(400MHz, DMSO-d_6_): 10.67 (s, 1H), 10.28 (s, 1H), 8.69 (s, 1H), 8.39 (s, 1H), 8.06 (d, J = 4.0Hz, 1H), 7.88 (d, J = 4.0Hz, 1H), 7.72 (d, J = 8.0Hz, 1H), 7.60 (t, J = 8.0Hz, 1H), 7.49–7.43 (m, 2H), 7.38–7.27 (m, 3H), 6.88 (d, J = 8.0Hz, 1H), 5.77 (s, 2H), 4.58 (s, 2H). ^13^C NMR(100MHz, DMSO-d_6_): 165.6, 164.9, 146.4, 143.5, 136.1, 135.1, 135.0, 133.4, 131.2, 131.1, 130.9, 129.5, 128.8, 128.5, 127.5, 123.6, 123.4, 122.8, 116.0, 114.9, 109.1, 67.3, 53.7.

3-(1-(4-bromo-2-fluorobenzyl)-1H-1,2,3-triazol-4-yl)-N-(3-oxo-3,4-dihydro-2H-benzo[b][1,4]oxazin-7-yl)benzamide (compound **f13**): Pure 96.6%. white solid, HR-MS(ESI): Calcd. C24H18BrFN5O3 [M+H]^+^ *m/z*: 522.0577, found: 522.0572. ^1^H NMR(400MHz, DMSO-d_6_): 10.67 (s, 1H), 10.27 (s, 1H), 8.71 (s, 1H), 8.38 (s, 1H), 8.06 (d, J = 4.0Hz, 1H), 7.88 (d, J = 8.0Hz, 1H), 7.63–7.36 (m, 8H), 6.88 (d, J = 8.0Hz, 1H), 5.72 (s, 2H), 4.58 (s, 2H). ^13^C NMR(100MHz, DMSO-d_6_): 165.6, 164.9, 146.5, 143.5, 136.1, 135.0, 133.0, 132.9, 132.8, 131.2, 129.5, 128.6, 128.5, 128.3, 127.5, 124.8, 123.6, 122.8, 122.6, 119.7, 119.5, 116.0, 114.9, 109.1, 67.3, 47.7, 47.2.

3-(1-(3-fluorobenzyl)-1H-1,2,3-triazol-4-yl)-N-(3-oxo-3,4-dihydro-2H-benzo[b][1,4]oxazin-7-yl)benzamide (compound **f14**): Pure 95.1%. white solid, HR-MS(ESI): Calcd. C24H19FN5O3 [M+H]^+^ *m/z*: 444.1472, found: 444.1463. ^1^H NMR(400MHz, DMSO-d_6_): 10.67 (s, 1H), 10.28 (s, 1H), 8.76 (s, 1H), 8.39 (s, 1H), 8.06 (d, J = 8.0Hz, 1H), 7.88 (d, J = 8.0Hz, 1H), 7.60 (d, J = 8.0Hz, 1H), 7.50–7.45 (m, 2H), 7.38 (dd, J1 = 4.0Hz, J2 = 4.0Hz, 1H), 7.25–7.17 (m, 3H), 6.88 (d, J = 8.0Hz, 1H), 5.72 (s, 2H), 4.58 (s, 2H). ^13^C NMR(100MHz, DMSO-d_6_): 165.6, 164.9, 163.8, 161.4, 146.6, 143.5, 139.0, 138.9, 135.0, 131.4, 131.3, 131.2, 129.5, 128.5, 127.5, 124.8, 124.5, 124.5, 123.6, 122.6, 116.0, 115.4, 115.2, 114.9, 109.1, 67.3, 52.9.

3-(1-(2-chloro-6-fluorobenzyl)-1H-1,2,3-triazol-4-yl)-N-(3-oxo-3,4-dihydro-2H-benzo[b][1,4]oxazin-7-yl)benzamide (compound **f15**): Pure 94.7%. white solid, HR-MS(ESI): Calcd. C24H18ClFN5O3 [M+H]^+^ *m/z*: 478.1082, found: 478.1069. ^1^H NMR(400MHz, DMSO-d_6_): 10.66 (s, 1H), 10.26 (s, 1H), 8.69 (s, 1H), 8.37 (s, 1H), 8.06 (d, J = 8.0Hz, 1H), 7.87 (d, J = 8.0Hz, 1H), 7.61–7.35 (m, 5H), 6.88 (d, J = 8.0Hz, 1H), 5.79 (s, 2H), 4.57 (s, 2H). ^13^C NMR(100MHz, DMSO-d_6_): 165.6, 164.9, 146.2, 143.5, 136.1, 135.0, 132.4, 132.3, 131.1, 129.4, 128.6, 127.5, 126.4, 124.9, 123.6, 122.6, 116.0, 115.6, 115.3, 114.9, 109.1, 67.2, 45.1.

N-(3-oxo-3,4-dihydro-2H-benzo[b][1,4]oxazin-7-yl)-3-(1-(4-(trifluoromethyl)benzyl)-1H-1,2,3-triazol-4-yl)benzamide (compound **f16**): Pure 97.6%. white solid, HR-MS(ESI): Calcd. C25H19F3N5O3 [M+H]^+^ *m/z*: 494.1440, found: 494.1418. ^1^H NMR(400MHz, DMSO-d_6_): 10.67 (s, 1H), 10.28 (s, 1H), 8.78 (s, 1H), 8.39 (s, 1H), 8.06 (d, J = 8.0Hz, 1H), 7.89 (d, J = 8.0Hz, 1H), 7.79–7.76 (m, 3H), 7.61–7.59 (m, 4H), 7.50 (s, 1H), 7.37 (d, J = 8.0Hz, 1H), 5.82 (s, 2H), 4.58 (s, 2H). ^13^C NMR(100MHz, DMSO-d_6_): 165.5, 164.9, 146.7, 143.5, 141.0, 136.1, 135.0, 131.2, 129.5, 129.4, 129.4, 129.1, 128.5, 127.5, 126.2, 126.2, 126.0, 126.0, 124.8, 123.6, 122.8, 116.0, 114.9, 109.1, 67.3, 53.2, 52.9.

3-(1-(3-bromobenzyl)-1H-1,2,3-triazol-4-yl)-N-(3-oxo-3,4-dihydro-2H-benzo[b][1,4]oxazin-7-yl)benzamide (compound **f17**): Pure 96.7%. white solid, HR-MS(ESI): Calcd. C24H19BrN5O3 [M+H]^+^ *m/z*: 504.0671, found: 504.0657. ^1^H NMR(400MHz, DMSO-d_6_): 10.67 (s, 1H), 10.28 (s, 1H), 8.76 (s, 1H), 8.39 (s, 1H), 8.07–8.04 (m, 1H), 7.90–7.87 (m, 1H), 7.62–7.56 (m, 3H), 7.50 (s, 4H), 7.38–7.37 (m, 3H), 6.88 (d, J = 8.0Hz, 1H), 5.70 (s, 2H), 4.58 (s, 2H). ^13^C NMR(100MHz, DMSO-d_6_): 165.6, 164.9, 146.6, 143.5, 138.9, 136.1, 135.0, 131.6, 131.5, 131.2, 129.5, 128.5, 127.5, 127.5, 124.8, 123.6, 122.67, 122.3, 116.0, 114.8, 109.1, 67.3, 52.7, 49.0.

3-(1-(4-chlorobenzyl)-1H-1,2,3-triazol-4-yl)-N-(3-oxo-3,4-dihydro-2H-benzo[b][1,4]oxazin-7-yl)benzamide (compound **f18**): Pure 95.8%. white solid, HR-MS(ESI): Calcd. C24H19ClN5O3 [M+H]^+^ *m/z*: 460.1176, found: 460.1152. ^1^H NMR(400MHz, DMSO-d_6_): 10.67 (s, 1H), 10.27 (s, 1H), 8.73 (s, 1H), 8.38 (s, 1H), 8.05 (d, J = 8.0Hz, 1H), 7.88 (d, J = 8.0Hz, 1H), 7.60 (t, J = 8.0Hz, 1H), 7.49–7.46 (m, 3H), 7.42–7.35 (m, 3H), 6.88 (d, J = 8.0Hz, 1H), 5.69 (s, 2H), 4.58 (s, 2H). ^13^C NMR(100MHz, DMSO-d_6_): 165.6, 164.9, 146.6, 143.5, 136.1, 135.3, 135.0, 133.4, 131.2, 130.4, 129.5, 129.3, 128.5, 127.5, 124.8, 123.6, 122.5, 116.0, 114.9, 109.1, 67.3, 52.8.

3-(1-(3-iodobenzyl)-1H-1,2,3-triazol-4-yl)-N-(3-oxo-3,4-dihydro-2H-benzo[b][1,4]oxazin-7-yl)benzamide (compound **f19**): Pure 97.1%. white solid, HR-MS(ESI): Calcd. C24H19IN5O3 [M+H]^+^ *m/z*: 552.0533, found: 552.0517. ^1^H NMR(400MHz, DMSO-d_6_): 10.67 (s, 1H), 10.28 (s, 1H), 8.76 (s, 1H), 8.38 (s, 1H), 8.05 (d, J = 8.0Hz, 1H), 7.88 (d, J = 8.0Hz, 1H), 7.79 (s, 1H), 7.41 (d, J = 8.0Hz, 1H), 7.60 (t, J = 8.0Hz, 1H), 7.50 (s, 1H), 7.38 (t, J = 4.0Hz, 2H), 7.21 (t, J = 4.0Hz, 2H), 6.88 (d, J = 8.0Hz, 1H), 5.66 (s, 2H), 4.58 (s, 2H). ^13^C NMR(100MHz, DMSO-d_6_): 165.6, 164.9, 146.6, 143.5, 138.8, 137.4, 137.0, 136.1, 135.0, 131.4, 131.2, 129.5, 128.5, 127.9, 127.5, 124.8, 123.6, 122.6, 116.0, 114.8, 109.1, 95.5, 67.3, 52.6.

3-(1-(2-methylbenzyl)-1H-1,2,3-triazol-4-yl)-N-(3-oxo-3,4-dihydro-2H-benzo[b][1,4]oxazin-7-yl)benzamide (compound **f20**): Pure 96.4%. white solid, HR-MS(ESI): Calcd. C25H22N5O3 [M+H]^+^ *m/z*: 440.1723, found: 440.1714. ^1^H NMR(400MHz, DMSO-d_6_): 10.66 (s, 1H), 10.26 (s, 1H), 8.63 (s, 1H), 8.37 (s, 1H), 8.06 (d, J = 4.0Hz, 1H), 7.86 (d, J = 8.0Hz, 1H), 7.58 (t, J = 8.0Hz, 1H), 7.48 (s, 1H), 7.35 (d, J = 8.0Hz, 1H), 7.27–7.16 (m, 4H), 6.87 (d, J = 8.0Hz, 1H), 5.68 (s, 2H), 4.57 (s, 2H). ^13^C NMR(100MHz, DMSO-d_6_): 165.6, 164.9, 146.4, 143.5, 136.8, 136.1, 135.0, 134.4, 131.3, 130.9, 130.9, 129.4, 129.2, 128.8, 128.5, 127.4, 126.8, 124.8, 123.6, 122.4, 116.0, 114.8, 109.1, 67.3, 51.7, 19.1.

### 6.2 Biological study

#### 6.2.1 Drug preparation and administration

Test compounds were dissolved in dimethyl sulfoxide (DMSO) at 10 mM as stock solution and diluted before *in vitro* drug administration. For the *in vivo* experiments, test compound were dissolved by 5% DMSO, 40% PEG300, 1% Tween 80 and saline for gavage administration.

#### 6.2.2 Cell culture

BV-2 cells (National Collection of Authenticated Cell Cultures, China). Cells were cultured in Dulbecco’s modified Eagle’s medium (LifeTech, Grand Island, NY, United States) supplemented with 10% heat-inactivated fetal bovine serum (Gibco, Grand Island, NY, United States) at 37°C in a humidified atmosphere containing 5% CO_2_.

#### 6.2.3 Griess assay

BV-2 cells were seeded at a density of 2 × 10^4^ cells per well in a 96-well culture plate and incubated in a humidified incubator with 5% CO_2_ at 37°C. After 24 h of incubation, 10 μM of the test compounds were added to the treatment group and incubated for 2 h. Subsequently, LPS was added to both the treatment group and the LPS model group to achieve a final concentration of 100 ng/mL. The cells were then incubated for another 24 h. After incubation, 50 µL of the supernatant from each well was mixed with 50 µL of Griess reagent and incubated at room temperature for 15 min. The optical density (OD) at 540 nm was measured using a microplate reader (Biotek).

#### 6.2.4 MTT assay

BV-2 cells were seeded at a density of 2 × 10^4^ cells per well in a 96-well culture plate and incubated in a humidified incubator with 5% CO2 at 37°C. After 24 h of incubation, 10 μM of the test compounds were added to the treatment group and incubated for 2 h. Subsequently, LPS was added to both the treatment group and the LPS model group to achieve a final concentration of 100 ng/mL. The cells were then incubated for another 24 h. Following this, 0.5 mg/mL MTT was added to each well for viable cell staining. After 1 h of incubation, the culture medium was discarded, and 100 µL of DMSO was added to each well. The plate was then shaken to fully dissolve the formazan crystals. The optical density (OD) at 490 nm was measured using a microplate reader (Biotek).

#### 6.2.5 Real-time PCR and transcriptome analysis

Total RNA was extracted using TRNzol (Tiangen Biotech Co., LTD., Beijing, China) following the manufacturer’s instructions. Complementary DNA (cDNA) was synthesized using the HiScript III RT SuperMix for qPCR (Vazyme Biotech Co., Ltd., Nanjing, China) with 500 ng of total RNA. Quantitative real-time PCR was performed using the ChamQ Pro Universal SYBR qPCR Master Mix (Vazyme Biotech Co., Ltd., Nanjing, China) on an ABI 7500 real-time PCR system (Applied Biosystems, Foster City, CA), according to the manufacturer’s instructions. The primers used are listed in Table 4. Relative gene expression was calculated using the ΔΔCT method.

**TABLE 4:** List of primers used in Real-Time PCR.

Gene	Forward primer (5′-3′)	Reverse primer (5′-3′)
IL-1β	GCA​ACT​GTT​CCT​GAA​CTC​AAC​T	ATC​TTT​TGG​GGT​CCG​TCA​ACT
IL-6	GGA​GGC​TTA​ATT​ACA​CAT​GTT	TGA​TTT​CAA​GAT​GAA​TTG​GAT
TNF-α	CCC​TCA​CAC​TCA​GAT​CAT​CTT​CT	GCT​ACG​ACG​TGG​GCT​ACA​G
iNOS	GTT​CTC​AGC​CCA​ACA​ATA​CAA​A	GTG​GAC​GGG​TCG​ATG​TCA​C
COX-2	TTG​AAG​ACC​AGG​AGT​ACA​GC	GGT​ACA​GTT​CCA​TGA​CAT​CG
GAPDH	GGT​GAA​GGT​CGG​TGT​GAA​CG	GGT​AGG​AAC​ACG​GAA​GGC​CA

#### 6.2.6 Western blot analysis

Whole cell extracts were prepared by lysing cells on ice for 30 min using RIPA lysis buffer containing 1 mM NaF, 1 mM Na3VO4, 1 mM PMSF, and 1% Protease Inhibitor Cocktail (Sigma, St. Louis, MO, United States). The lysates were centrifuged at 12,000 g for 15 min at 4°C, and the supernatants were collected. Protein concentrations were measured using the BCA protein assay (Thermo Scientific, Rockford, IL, United States) according to the manufacturer’s instructions.

Western blotting was performed based on previously described methods. Equal amounts of protein were separated by sodium dodecyl sulfate polyacrylamide gel electrophoresis (SDS-PAGE) and transferred onto 0.2 μm nitrocellulose membranes (Whatman, Maidstone, Kent, United Kingdom). The membranes were blocked in 5% skimmed milk in Tris-buffered saline with Tween 20 (TBST; 50 mM Tris base, 150 mM NaCl, 0.05% v/v Tween 20, pH 7.4) for at least 1 h and then incubated with primary antibodies overnight at 4°C. Following incubation, the blots were washed three times with TBST buffer and then incubated with horseradish peroxidase-conjugated secondary antibodies (1:5,000, Kangchen Biotechnology, Shanghai, China) for 1 h at room temperature. The blots were developed using an enhanced chemiluminescence reagent (Millipore Corporation, Bedford, MA, United States) after three additional washes with TBST buffer. Protein band intensities were analyzed using ImageJ software.

The primary antibodies used were as follows: anti-COX-2 (1:1,000, Cell Signaling Technology, Beverly, MA, United States), anti-iNOS (1:500, BD Biosciences, San Jose, CA, United States), anti-Nrf2 (1:1,000, Proteintech, Wuhan, China), anti-HO-1 (1:5,000, Proteintech, Wuhan, China), and anti-β-actin (1:10,000, Sigma-Aldrich, St. Louis, MO, United States).

#### 6.2.7 Cellular ROS level measurement

BV-2 microglia cells were seeded at a density of 1 × 10^5^ cells per well in a 12-well culture plate. After treatment with LPS for 18 h, the cells were collected by trypsin digestion. 1 mL of DCFH-DA staining solution was added and incubated at 37°C for 30 min. The cells were washed twice with 1 × PBS and mean fluorescence intensity (MFI) was detected using a flow cytometer (BD Accuri™ C6, United States).

#### 6.2.8 Acute toxicity test

Kunming mice (half male and half female, 8 weeks old) were randomly allocate into 4 groups: e16-treated group (male), e16-treated group (female), Vehicle group (male) and Vehicle group (female). The Vehicle groups were given corresponding solvents, while the e16 groups were given the e16 solution (250 mg/kg, respectively). Body weight and appearance were monitored for a total period of 15 days (from the first day after treating with e16). The mice were killed on the 15th day, and blood samples were collected for the biochemistry test. Then, we measured liver and kidney function indicators such as alanine aminotransferase (ALT) and aspartate aminotransferase (AST) (Nanjing Jiancheng Bioengineering Institute, China). The organs (heart, liver, spleen, lungs, kidneys) were harvested, weighed and fixed in 10% formaldehyde and paraffin-embedded for histological examination. Finally, these sections were stained with haematoxylin and eosin for light microscopic examination.

#### 6.2.9 Statistical analyses

Data were presented as means ± SEM and performed using Graph Prim 7.0. A two-tailed Student’s *t*-test or one-way analysis of variance followed by a Student-Newman-Keuls (SNK) test were used to assess significant differences. *P* < 0.05 was considered statistically significant.

#### 6.2.10 Molecular docking methods

The X-ray crystal structure of mouse Keap1 [PDB ID: 1X2R (Padmanabhan et al., 2006)] which possesses an Nrf2 binding site was retrieved from the Protein Data Bank (https://www.rcsb.org) with a resolution value of 1.70 Å. The initial structure was processed using Protein Preparation Wizard in Schrödinger (Sastry et al., 2013). The chemical structures of e2, e16, and e20 were prepared using the LigPrep module (Schrödinger, 2021). The Nrf2 binding site was defined as the docking area by using the Receptor Grid Generation tool, and the Grid file was used to dock ligands into receptor protein to identify the potential binding modes (Friesner et al., 2004).

## Data Availability

The datasets presented in this study can be found in online repositories. The names of the repository/repositories and accession number(s) can be found in the article/Supplementary Material.
